# Disruption of KDM4C-ALDH1A3 feed-forward loop inhibits stemness, tumorigenesis and chemoresistance of gastric cancer stem cells

**DOI:** 10.1038/s41392-021-00674-5

**Published:** 2021-09-22

**Authors:** Tingyuan Lang, Jia Xu, Lei Zhou, Zhiqi Zhang, Xinli Ma, Jiayi Gu, Jingshu Liu, Yunzhe Li, Dongyan Ding, Jiangfeng Qiu

**Affiliations:** 1 Department of Gynecologic Oncology, Chongqing University Cancer Hospital, School of Medicine, Chongqing University, Chongqing, People’s Republic of China; 2 Department of Gastrointestinal Surgery, Renji Hospital Shanghai Jiao Tong University School of Medicine, Shanghai, People’s Republic of China; 3 Singapore Eye Research Institute, The Academia, Singapore, Singapore; 4 Department of Ophthalmology, Yong Loo Lin School of Medicine, National University of Singapore, Singapore, Singapore; 5 Ophthalmology and Visual Sciences Academia Clinical Program, Duke-NUS Medical School, Singapore, Singapore; 6 Department of General Surgery, Shanghai Fourth People’s Hospital Affiliated to Tongji University School of Medicine, Shanghai, People’s Republic of China

**Keywords:** Cancer stem cells, Gastrointestinal cancer

**Dear Editor**,

Gastric cancer (GC) is a considerable global health burden; the median survival of advanced GC is less than 1 year.^1^ Cancer stem cells (CSCs), a small population of cancer cells with stem cell-like properties, are the major cause of treatment failure, including GC,^2^ however, the mechanisms underlying stemness maintenance of GC stem cells (GCSCs) are still poorly understood.

KDM4C, also known as JMJD2C/GASC1, is a member of Jumonji domain-2 family histone demethylases, which was first implicated as an oncogene that is amplificated in the KYSE-150 esophageal cancer cell line.^3^ Dysregulation of KDM4C tightly links with tumorigenesis in several types of cancer; epigenetic regulation of gene expression is the prime mechanism.^4^ KDM4C also plays important role in embryonic stem cells and CSCs.^3,4^ KDM4C inhibitors, such as SD70, have been developed for anticancer therapy.^3,4^ However, the role of KDM4C in GC is underestimated due to a lack of evidence for its dysregulation in normal GC cells. Our result from the immunohistochemistry study with clinical samples confirmed this point (Fig. 1a and Supplementary Fig. S1a). However, in poorly differentiated GC tissues, the expression of KDM4C was significantly upregulated (Fig. 1a) and positively correlates with GCSCs markers (Fig. 1b and Supplementary Fig. S1b). Furthermore, high expression of KDM4C predicts poor prognosis of patients with poorly differentiated GC (Fig. 1c), which indicated the potential role of KDM4C in GCSCs. On the other hand, elevated activity of aldehyde dehydrogenase (ALDH) is one of the most important characteristics of GCSCs; ALDH family members have been recognized as CSCs markers in various solid neoplasms and play crucial roles through metabolic signalings, such as ALDH-dependent retinoic acid (RA) signaling.^5^ Thus, elucidating the upstream regulatory mechanisms of ALDH family members is significant to develop therapeutic strategies targeting GCSCs. By proteomics study, pathway analysis, and Q-PCR (quantitative real-time PCR) verification, we found that ALDH1A3 was upregulated in KDM4C-overexpressing GC cells (Supplementary Fig. S2 and Fig. S3), indicating the potential link between KDM4C and ALDH1A3. Given their important roles and our previous observation, in this study, we investigated the effect of KDM4C and ALDH1A3 in GCSCs and their potential use as therapeutic targets.

**Fig. 1:**
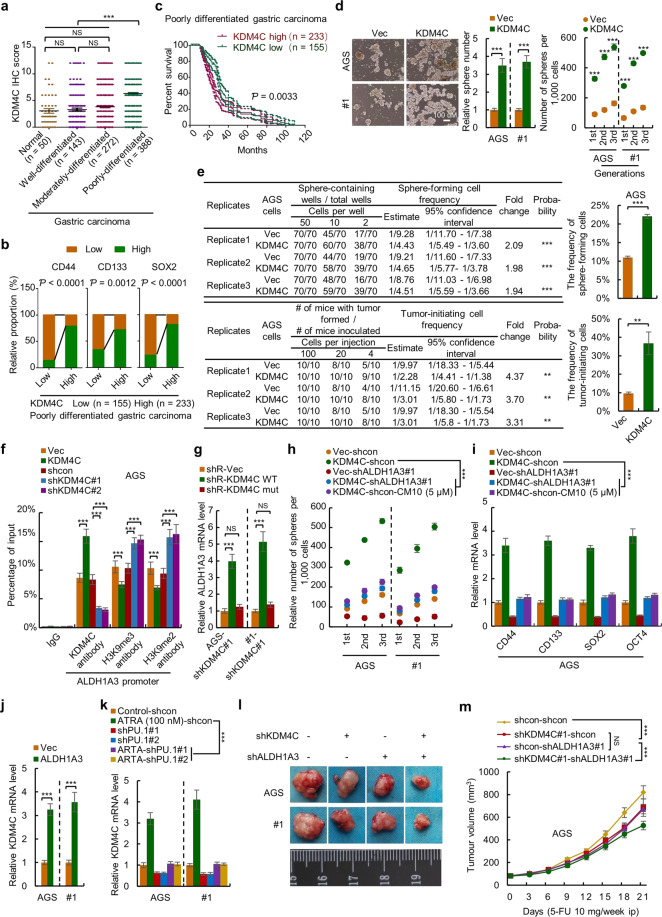
Disruption of KDM4C-ALDH1A3 feed-forward loop inhibits stemness, tumorigenesis, and chemoresistance of gastric cancer stem cells. **a** The scores of KDM4C immunohistochemical staining in 50 normal, 143 well-differentiated, 272 moderately differentiated, and 388 poorly differentiated tumor tissues. The data were analyzed by one-way ANOVA. Error bars indicate the standard error of the mean (SEM). **b** The correlations of KDM4C with CD44, CD133, and SOX2 were determined by Spearman correlation analysis. **c** The association of KDM4C expression with overall survival of poorly differentiated gastric cancer patients was analyzed by Kaplan-Meier analysis and a 95% confidence interval was shown. **d** KDM4C promotes the sphere-forming capacity of gastric cancer cells on serial passage. The sphere number of indicated cells at the density of 3000 cells/well was recorded (left). The sphere number of primary, secondary, and tertiary passaged KDM4C-overexpressing and control indicated cells were counted (right). The data were analyzed by Student’s *t-*test. Error bars indicate standard deviation (SD) (*n* = 3). **e** The in vitro sphere-forming (top) and in vivo tumor-initiating (bottom) frequency of KDM4C-overexpressing and control gastric cancer cells were determined by limiting dilution assay. Error bars indicate SD (Student’s *t-*test, *n* = 3). **f** The binding between KDM4C and the promoter of ALDH1A3 and the effect of KDM4C on H3K9me and H3K9m2 levels at ALDH1A3 promoter were analyzed by chromatin immunoprecipitation (ChIP) assay. Error bars indicate SD (Student’s *t-*test, *n* = 3). **g** The mRNA levels of ALDH1A3 in KDM4C-knockdown AGS cells transfected with shRNA-resistant H190A/E912A mutant (demethylase dead) KDM4C and shRNA-resistant wild-type KDM4C were determined by quantitative real-time PCR (Q-PCR). Error bars indicate SD (one-way ANOVA, *n* = 3). **h**, **i** The serial sphere-forming capacity (**h**) and the mRNA levels of gastric cancer stem cell markers (**i**) in KDM4C-overexpressing cells with ALDH1A3 depletion or ALDH1A3 inhibitor treatment and control cells were determined by sphere formation assay (**h**) and Q-PCR (**i**). Error bars indicate SD (One-way ANOVA, *n* = 3). **j** Q-PCR analysis of KDM4C mRNA level in ALDH1A3-overexpressing indicated cells. Error bars indicate SD (Student’s *t-*test, *n* = 3). **k** Q-PCR analysis of KDM4C mRNA levels in ATRA-treated, ATRA-treated PU.1-knockdown, and corresponding control cells. Error bars indicate SD (One-way ANOVA, *n* = 3). **l** Inhibition of KDM4C and ALDH1A3 synergistically inhibits tumorigenesis of sphere-derived gastric cancer cells. The tumor volume in mice bearing KDM4C-knockdown, ALDH1A3-knockdown, a combination of KDM4C-knockdown and ALDH1A3-knockdown, and control sphere-derived gastric cancer cells was measured in indicated days (*n* = 7). **m** Inhibition of KDM4C and ALDH1A3 synergistically sensitizes gastric cancer cells to chemotherapeutic drugs in vivo. The tumor volume in 5-FU-treated mice bearing indicated gastric cancer cells was measured in indicated days (*n* = 9). Error bars indicate SD (one-way ANOVA). ip: intraperitoneal injection. *Ƥ < 0.05, **Ƥ < 0.01, ***Ƥ < 0.001

To confirm the role of KDM4C, we investigated the KDM4C expression level in GCSCs by Q-PCR. KDM4C is significantly upregulated in suspension-cultured spheres, CD44+ and ALDH+ cells, compared to adherent-cultured, CD44− and ALDH− cells (Supplementary Fig. S4a). KDM4C overexpression (Supplementary Fig. S4b) enhanced the stemness of both cell line and primary GC cells reflected by the increased serial sphere-forming capacity (Fig. 1d), expression of GCSCs markers (CD44, CD133, SOX2, OCT4) (Supplementary Fig. S4c), ALDH activity (Fig. S4c), the frequency of sphere-forming and tumor-initiating cells (Fig. 1e), and multi-generational tumorigenicity (Supplementary Fig. S4d). Knockdown of KDM4C or treatment with KDM4C inhibitor significantly inhibited the stemness of GC cells, including serial sphere-forming capacity, markers expression, ALDH activity, and the frequency of sphere-forming cells (Supplementary Fig. S5a–g). These results demonstrated that KDM4C promotes the stemness of GC cells.

We next investigated the role of ALDH1A3 in GCSCs. Similar results were obtained; ALDH1A3 is aberrantly upregulated in moderately and poorly differentiated GC tissues (Supplementary Fig. S6a), positively correlates with GCSCs markers (Supplementary Fig. S6b), and predicts poor prognosis (Supplementary Fig. S6c). Ectopic expression of ALDH1A3 increased the serial sphere-forming capacity, the expression of GCSCs markers, ALDH activity, the frequency of sphere-forming and tumor-initiating cells, and multiple-generational tumorigenicity (Supplementary Fig. S6d–h) in GC cells. Knockdown of ALDH1A3 and treatment with ALDH1A3 inhibitor, CM10, inhibited the serial sphere-forming capacity, the expression of GCSCs markers, and ALDH activity (Supplementary Fig. S7a–e). These results demonstrated that ALDH1A3 positively regulates GC stemness.

As KDM4C is a histone demethylase,^3,4^ we examined whether KDM4C-induced histone demethylation involves in the regulation of GC stemness and ALDH1A3 transcription. The positive correlation between KDM4C and ALDH1A3 was found in poorly differentiated GC tissues (Supplementary Fig. S8a). KDM4C positively regulated the transcription of ALDH1A3 as revealed by Q-PCR, unclear run-on and luciferase reporter assay (Supplementary Fig. S8b, c), and no obvious effect of KDM4C on the stability of ALDH1A3 protein was observed (Supplementary Fig. S8d). Results from the ChIP (Chromatin immunoprecipitation) assay showed that KDM4C directly bound to ALDH1A3 promoter and negatively regulated H3K9me2 and H3K9me3 levels at ALDH1A3 promoter (Fig. 1f). Only shRNA resistant wild-type KMD4C, but not demethylase dead mutant (H190A and E912A) KMD4C, recovered the ALDH1A3 mRNA level (Fig. 1g) and the stem-like properties (Supplementary Fig. S8e–g) in KDM4C-knockdown cells. Furthermore, depletion of ALDH1A3 or ALDH1A3 inhibitor treatment significantly abolished the effect of KDM4C on serial sphere-forming capacity, the expression of GCSCs markers, and ALDH activity of GC cells (Fig. 1h, i and Supplementary Fig. S9). These results demonstrated that KDM4C epigenetically activates ALDH1A3 transcription through histone demethylation and this process is critical for KDM4C-induced GC stemness maintenance.

We next found that the activated ALDH1A3 in turn activates KDM4C. Similarly, ALDH1A3 upregulated the transcription of KDM4C (Fig. 1j and Supplementary Fig. S10a), and no effect of ALDH1A3 on KDM4C stability was observed (Supplementary Fig. S10b). There is no direct binding between ALDH1A3 and the KDM4C promoter was detected (Supplementary Fig. S10c). Treatment with ALDH1A3 product all-trans retinoic acid (ATRA), a nuclear receptor with transcriptional regulatory property, obtained a similar increase in KDM4C transcription (Supplementary Fig. S10d) and this effect was abolished by ablation of PU.1 (an ATRA receptor) (Fig. 1k and Supplementary Fig. S10e, f). Furthermore, KDM4C depletion or KDM4C inhibitor treatment significantly abolished the effect of ALDH1A3 on GC stemness (Supplementary Fig. S11). These results demonstrated that ALDH1A3 transcriptionally activates KDM4C by RA signaling to support GC stemness and the above results revealed an important feedforward mechanism, the KDM4C-ALDH1A3 loop, for GC stemness maintenance.

We next investigated whether the KDM4C-ALDH1A3 loop can be served as a drug target for eradicating GCSCs. We found that ablation of both KDM4C and ALDH1A3 reduced the volume of tumor formed by sphere-derived GC cells and the expression of GCSCs markers of the tumor cells, and simultaneous depletion led to a synergistic effect in mice (Fig. 1l and Supplementary Fig. S12a, b). Treatment with inhibitors obtained similar results (Supplementary Fig. S12c). As CSCs are resistant to chemotherapeutic drugs,^1^ we next examined the sensitizing effect of KDM4C and ALDH1A3 inhibition. The resistance of sphere-derived GC cells was confirmed in vitro (Supplementary Fig. S13a), and overexpression of KDM4C and ALDH1A3 produced a similar resistance (Supplementary Fig. S13b). As expected, both knockdowns of KDM4C and ALDH1A3 and treatment with inhibitors sensitized the sphere-derived cells to 5-FU and cisplatin in vitro (Supplementary Fig. S13c) and in vivo (Fig. 1m and Supplementary Fig.S13d), and simultaneous inhibition produced a synergistic effect (Supplementary Figs. S13c, 1m, and S13d). Furthermore, knockdown of KDM4C and ALDH1A3 prolonged the survival of tumor-bearing mice (Supplementary Fig. S13e).

In summary, this study revealed an important feedforward mechanism, the KDM4C-ALDH1A3 loop, for GC stemness maintenance, which would be served as a novel therapeutic target for eradicating human GCSCs.

## Supplementary information

Supplementary materials

## Data Availability

All mass spectrometry proteomics data have been deposited to the ProteomeXchange Consortium via the PRIDE partner repository with the data set identifier PXD023455. Other source data and reagents are available from the corresponding author upon reasonable request.

## References

[1] Cutsem EV, Sagaert X, Topal B, Haustermans K, Prenen H (2016). Gastric cancer. Lancet.

[2] Lytle NK, Barber AG, Reya T (2018). Stem cell fate in cancer growth, progression and therapy resistance. Nat. Rev. Cancer.

[3] Michalak EM, Burr ML, Bannister AJ, Dawson MA (2019). The roles of DNA, RNA and histone methylation in ageing and cancer. Nat. Rev. Mol. Cell Biol..

[4] Metzger E (2017). KDM4 inhibition targets breast cancer stem-like cells. Cancer Res..

[5] Raha D (2014). The cancer stem cell marker aldehyde dehydrogenase is required to maintain a drug tolerant tumor cell subpopulation. Cancer Res..

