# Supplementary material for: Disruption of KDM4C-ALDH1A3 feed-forward loop inhibits stemness, tumorigenesis and chemoresistance of gastric cancer stem cells
Source: Signal Transduct Target Ther. 2021 Sep 22;6:336. doi: 10.1038/s41392-021-00674-5 (PMC8455594; doi:10.1038/s41392-021-00674-5)
Supplement: Supplementary file 1 — Supplementary materials [file 41392_2021_674_MOESM1_ESM.docx]

**Supplemental Material for**

**Disruption of KDM4C-ALDH1A3 feed-forward loop inhibits stemness, tumorigenesis and chemoresistance of gastric cancer stem cells**

**Tingyuan Lang^1#^, Jia Xu^2#^, Lei Zhou^3,4,5^, Zhiqi Zhang^6^, Xinli Ma^2^, Jiayi Gu^2^, Jingshu Liu^1^, Yunzhe Li^1^, Dongyan Ding^1^, Jiangfeng Qiu^2*^**

^#^TY.L and J.X. contributed equally to this work.

^*^To whom correspondence may be addressed. Email: [qjf0228@126.com](mailto:qjf0228@126.com).

**This file includes:**

Materials and Methods

Tables. S1 and S2

Figures. S1 to S13

**Materials and Methods**

**Cell lines and cell culture**

Human gastric cancer (GC) cell lines AGS, NCI-N87, SNU-16 were purchased from American Type Culture Collection (Manassas, Virginia, USA). MKN45 human GC cell line was purchased from Cell bank of Chinese Academy of Sciences (Shanghai, China). AGS cells were cultured in F-12K cell culture medium. NCI-N87, MKN45 and SUN-16 cells were cultured in RPMI-1640 medium. The complete growth medium was made by supplement of 10% fetal bovine serum (FBS) and antibiotic cocktail. All components for cell culture were purchased from Thermo Fisher (Waltham, MA, USA). The cells were cultured at 37 °C in 5% CO2. All cells were validated by short tandem repeat profiling and the absence of mycoplasma was examined.

**Primary GC cell culture**

Tumour samples from GC patients were collected undergoing surgical gastrectomy. All samples were obtained from Renji Hospital Shanghai Jiao Tong University School of Medicine and all procedures were performed in accordance with the Institutional Review Board of Renji Hospital Shanghai Jiao Tong University School of Medicine (Shanghai, China). The specimens were maintained in transport medium (serum-free RPMI1640 medium supplemented with 2 μg ml^-1^ amphotericin B) during transport. The fat, connective and necrotic sections of the tumour sample were carefully removed. The remaining tissues were cut into small pieces (1 mm^3^) and placed into fibronectin-coated culture dish containing complete primary culture medium (RPMI1640 supplemented with 20% FBS and antibiotics cocktail) and incubated at 37 °C in 5% CO_2_. After attachment of the tissues (24-48 h), the medium changed every 24 h until the GC cells migrated and attached to the dish. The tissues were removed from the dish and the cells were digested and collected by centrifugation, followed by culture with a new dish. GRN and CA724 were used to identified the GC cells.

**Plasmids, primers, antibodies and reagents**

Lentivirus plasmid pCDH-CMV-MCS-EF1-Puro was kindly provided by professor Hongbin Ji (Shanghai Institutes for Biological Sciences, Chinese Academy of Sciences, Shanghai, China). KDM4C- and ALDH1A3-pCDH-CMV-MCS-EF1-Puro plasmids were produced by inserting the coding sequence region of KDM4C and ALDH1A3, which were amplified by PCR with genomic DNA of AGS, into pCDH-CMV-MCS-EF1-Puro plasmids, respectively. shRNA-resistant KDM4C was made by introducing non-sense mutations in shRNA-targeting sites. shRNA-resistant H190A/E912A mutant KDM4C were constructed by Q5 sit-directed mutagenesis kit (NEB, lpswich, MA, USA). shRNA lentivirus particles TRCN0000022057 and TRCN0000022054 for KDM4C; TRCN0000027212 and TCRN0000027183 for ALDH1A3; TRCN0000417534 and TRCN0000426240 for PU.1; control plasmids (SHC016) were purchased from Sigma-Aldrich (St. Louis, MO, USA); pGL4.20 from Promega (Madison, WI, USA) was used for luciferase reporter assay. The promoter region of ALDH1A3 and KDM4C was cloned into pGL4.20, respectively, for examination of the transcriptional activity of ALDH1A3 and KDM4C promoters. The information of primers and antibodies were included in Table S1 and S2. Fluorouracil (5-FU) and cisplatin were purchased from Selleckchem (Houston, TX, USA). 5-FU was resolved in ddH2O (for in vitro assay) and normal saline (for in vitro assay). Cisplatin was resolved in dimethylformamide (DMF) for both in vitro and in vivo assay. ATRA was obtained from Sigma-Aldrich (St. Louis, MO, USA). ALDH1A3 inhibitor CM10 was purchased from MedChem Express (Shanghai, China) and KDM4C inhibitor SD70 was obtained from Sigma-Aldrich (St. Louis, MO, USA). Both CM10 and SD70 were resolved in DMSO. SD70 and CM10 was administered by intraperitoneal injection at 10mg/kg and 20mg/kg once per day in xenograft model. All other reagents, except where indicated, were obtained from Sigma-Aldrich (St. Louis, MO, USA).

**Lentivirus packaging and transfection**

The recombinant plasmid, pCMV-dR8.2 and pCMV-VSVG were co-transfected into HEK293T cells for lentivirus packaging. The virus particles in culture medium were harvested after 24 h transfection. The medium containing virus particles were used for infection of target cells, followed by puromycin selection. The stable cells were verified by western blot.

**Sphere formation assay**

The cells were seeded at a density of 5 × 10^5^ cells per dish in 100 mm cell culture dish. After reaching 90% confluence, the cells were digested and collected, followed by culture in ultra-low attachment 6 well plate (Sigma-Aldrich, St. Louis, MO, USA) containing serum-free DMEM/F12 medium supplemented with 20 ng ml^-1^ recombinant human epidermal growth factor (rh-EGF, Thermo Fisher, Waltham, MA, USA), 20 ng ml^-1^ recombinant human basic fibroblast growth factor (rh-bFGF, Thermo Fisher, Waltham, MA, USA), 0.5 μg ml^-1^ glucocorticoid and 2% B27 at a density of 1 × 10^4^ cells per well. The spheres were formed after 15 days culture.

**CD44+ and ALDH+ cells isolation and ALDH activity evaluation**

Magnetic-activated cell sorting (MACS) was employed for isolation of CD44+ cells. CD44-magnetic beads were produced by incubating 10 μl streptavidin-coated superparamagnetic polystyrene beads (4 × 10^5^ beads ml^-1^, Thermo Fisher, Waltham, MA, USA) and 1 μg biotinylated anti-CD44 antibody (Miltenyi Biotec, Gladbach, Germany) in 1 ml PBS overnight at 4 °C, followed by centrifugation. The supernatant was then removed and the cells were added to CD44-magnetc beads at a ratio of 1:2 (cell number / bead number). After incubation at 4 °C for 1h, the CD44+ cells were separated by magnetic sorting.

ALDEFLUOR™ Kit (Stem Cell Technologies, Durham, NC, USA) was used to isolate cells with high ALDH (ALDH+ cells). The cells freshly dissociated from normal cultured cells were suspended in ALDH substrate (BAAA, 1 μM per 1 × 10^6^ cells) contained ALDEFLUOR assay buffer and incubated at 37 °C for 40 min. Cells treated with 50 mM of diethylaminobenzaldehyde (DEAB), a specific ALDH inhibitor, were used as negative control. A FACS Cell Sorter Flow Cytometer (BD Biosciences, Franklin Lakes, NJ, USA) was used to determine the intensity of fluorescence. ALDH+ and ALDH- cells were collected for ALDH1A3 mRNA level analysis.

**Western blot**

Total protein was isolated by RIPA buffer (Thermo Fisher, Waltham, MA, USA) containing a protease inhibitor cocktail (Thermo Fisher, Waltham, MA, USA). The samples were then separated by standard 8–12% sodium dodecyl sulfatepolyacrylamide gel electrophoresis and transferred to polyvinylidene fluoride (PVDF) membranes. The membranes were blocked by 5% defatted milk, followed by incubation with primary antibodies overnight at 4 °C. The corresponding secondary antibodies were then hybridized with samples in the membranes at room temperature for 2-4 h. The signals were visualized using enhanced chemiluminescence (Thermo Fisher, Waltham, MA, USA). The antibodies used were listed in Table S2.

**Quantitative Real-time polymerase chain reaction (Q-PCR)**

Total RNA isolation was performed by Trizol reagent (Thermo Fisher, Waltham, MA, USA) according to standard protocol. After removing of ethanol and residual DNA by evaporation and Turbo DNAse (Thermo Fisher, Waltham, MA, USA), the concentration of mRNA was determined by Nanodrop spectrophotometer (Thermo Fisher, Waltham, MA, USA). TaqMan RNA-to-Ct 1-Step Kit (Thermo Fisher, Waltham, MA, USA) was used to perform qRT-PCR assay on StepOne Real-Time System (Applied Biosystems, Thermo Fisher, Waltham, MA, USA). *GAPDH* was employed for normalization control. ∆∆Ct method was used for determination of relative mRNA level. The information of primers was provided in supplementary materials.

**Cell derived xenograft (CDX) model**

CDX model was performed with 6-8 weeks old female BALB/c nude mice (Pusheng Technology, Nanjing, China). All the procedures were approved by Institutional Animal Care and Use Committee in Renji Hospital Shanghai Jiao Tong University School of Medicine (Shanghai, China). The cells dissociated from suspension-cultured spheres were resuspended in RPMI1640 culture medium containing 25% Matrigel (Thermo Fisher, Waltham, MA, USA). Then the cells were incubated into the flank of the mice. The number of cells inoculated is based on the experimental design. The tumour was observed every 7 days.

**Immunohistochemical scoring method**

The gastric cancer specimens were provided by department of Gastrointestinal Surgery, Renji Hospital Shanghai Jiao Tong University School of Medicine. The immunohistochemical staining was performed according to standard protocol. The protein expression was assessed on a scale from 0-4 (<10% positive cells, 10-25% positive cells, 25-50% positive cells, 50-75% positive cells, >75% positive cells). The intensity of protein expression was scored on a scale from 0 to 3 (no staining, weak staining, moderate staining and strong staining). The percentage and intensity scores were combined according to the immunoreactive score (IRS) scoring method.^1^ The IRS is calculated as follows: Final expression score = percentage of positive cells multiplied by the staining intensity. This results in a final score ranging from 0-12. The final expression score was divided into 4 categories (0-1 = no expression, 2-3 = weak expression, 4-8 = moderate expression, 9-12 = strong expression).

**Limiting dilution assay (LDA) assay**

The cells were first cultured in suspension environment for sphere formation. Then, the cells were dissociated and collected, followed by seeding into ultra-low attachment 96-well culture plate (Corning, NY, USA) containing serum-free medium containing growth factors as mentioned above at a density of 50, 10 or 2 cells per well. The sphere-containing wells were counted after 2 weeks culture. The frequency of sphere-forming cells was determined by ELDA software (http://bioinf.wehi.edu.au/software/elda/)

To perform LDA assay in vivo, 100, 20, 4 cells were suspended in RPMI1640 culture medium containing 25% gel and inoculated into the flank of mice. The tumour for each mice was observed at the 45^th^ day after inoculation. ELDA software was employed to calculated the frequency of tumour-initiating cells.

**iTRAQ-based quantitative proteomic analysis**

The overall experiment design of the quantitative proteomic analysis is given in Fig. S2a. Briefly, KDM4C-overexpressing and control AGS cells were cultured in ultra-low attachment plate for sphere formation. The spheres were collected by centrifugation and 1 × 10^7^ digested cells were lysed by a mixture of acetonitrile and 50 mM ammonium bicarbonate (1:9) containing 0.1% (w/v) ProteaseMax powder (Promega). The lysate was then centrifuged (14,000 rpm, 7 min, room temperature) and the total protein concentration in the supernatant was measured. An aliquot of 100 μg protein for each sample was used for iTRAQ analysis as shown in Fig. S2b. The protein samples were incubated with 50 mM TCEP for 1 h at 60 °C and transferred to a centrifuge tube with a 30 kDa cut-off filter membrane and the alkylation (15mM IAA for 30 min at room temperature in dark) and digestion were performed on the membrane. After 3 times washes with 75% urea solution, the samples were incubated with 0.1 M Triethylammonium bicarbonate solution (TEAB). After digestion with trypsin (1:50) for 16 h at 37 °C, the peptides were eluted with a mixture of 0.1 M TEAB and 0.5 M sodium chloride. The peptides were dried and incubated with iTRAQ labeling reagents for 2 h at room temperature according to the manufacturer’s protocol (AB SCIEX). All labeled samples were then pooled for subsequent high pH fractionation.

High pH reverse phase (RP) fractionation was performed using a Waters HPLC system (Elstree, UK). Briefly, the samples were reconstituted in mobile phase A and injected into the system. Waters Acquity UPLC BEH C18, 3.5 μm, 3.0 × 150 mm was used for the peptide separation at the flow rate of 0.4 mL/min. Other LC conditions were as follow: mobile phase A - 20 mM ammonium formate (pH 10); mobile phase B - 80% Acetonitrile in 20 mM Ammonium formate (pH 10); gradient - 5-15% in 20 min, 15-40% in 20 min, 40–80% in 1 min. A total of 10 fractions were collected for further nano-LC-MS/MS analysis.

A Ultimate 3000 nanoLC system (Thermo Fisher Scientific) coupled with a TripleTOF 5600 (AB Sciex) was used for the nano-LC-MS/MS analysis. Two technical runs were performed for each fraction. Acclaim PepMap RSLC C18, 15 cm × 75 µm was used as the analytical column at the flow rate of 300 nl/min. Mobile phase consists of (A) 0.1% formic acid, 2% acetonitrile in water and mobile phase (B) 2/98 v/v of water/ACN with 0.1% formic acid. A 120-min gradient was used at the following settings: 5–12% in 30 min, 12–40% in 60 min, and 40–90% in 20 min.

The instrumental settings for the TripleTOF 5600 mass spectrometer were as follows: ISVF (Ionspray Voltage Floating) = 2000 V, CUR (curtain gas) = 30, GS2 (Ion source gas 1) = 10, IHT (Interface Heater Temperature) = 125, DP (declustering potential) = 100 V, NC (Nebuliser current) = 3 for nitrogen gas. IDA (information-dependent acquisition) mode. The data was acquired using Analyst TF 1.7 software (AB SCIEX). Other settings for IDA experiments were as follows: TOF mass survey scan - 0.25 s; mass range - 400–1250; product ion scan - 0.05 s; mass range: 100-1500; charge state = 2~5; maximum number of candidate ions to monitor per cycle = 40 spectra; abundance threshold >120 counts; the exclusion of former target ions: 12 s. IDA Advanced settings such as ‘dynamic accumulation’, ‘adjust CE when using iTRAQ Reagent’ and ‘rolling collision energy (CE)’ was included.

Data analysis was performed using ProteinPilot 5.0 software (AB SCIEX) and the protein database (version: uniprot_all_Oct2014) base on typical settings provided previously. Protein identification was performed by ProteinPilot software. Relative quantitation was performed with Pro Group algorithm in ProteinPilot base on peak areas of reporter ions. The data analysis strategy considering biological and technical replicates shown in Fig. S2a-c.

**Bioinformatics analysis**

iPathwayGuide online software was used to perform Gene ontology (GO) and pathway analysis of differential expressed proteins identified by proteomic analysis.

**Nascent RNA quantification**

The synthesis of KDM4C and ALDH1A3 was measured using Click-iT™ Nascent RNA Capture Kit (Thermo Fisher, Waltham, MA, USA). Briefly, the cells were cultured in 6-well plate at 30% confluence. After attachment, the cells were washed with PBS and incubated in medium containing 0.5 mM 5-ethynyl uridine (EU) for 1 h. After incubation, the total RNA of the cells was isolated with Trizol reagent. EU-labeled RNA was mixed with biotin azide (10 ug RNA : 1 mM Biotin Azide) for biotinylation, followed by purification with a streptavidin-coupled magnetic beads. The nascent mRNA levels of KDM4C and ALDH1A3 were measured by qRT-PCR assay.

**Dual-Luciferase Reporter Assay**

To determine the transcriptional activities of KDM4C and ALDH1A3 promoters, the promoter segments spanning -1200 to +115 bp relative to the transcription start site of KDM4C and ALDH1A3 were cloned into PGL4.20 plasmid. The recombinant plasmid and pRL-TK plasmid were co-transfected into the target cells by Lipofactamin 2000 (Thermo Fisher, Waltham, MA, USA). Dual-Luciferase Reporter Assay System (Promega Corporation, Madison, WI, USA) was used to measure the luciferase activity. Renilla luciferase activity was used as internal control.

**Chromatin immunoprecipitation (ChIP) assay**

The protein-DNA binding was examined by ChIP assay, which was performed using MAGnify™ Chromatin IP System (Thermo Fisher, Waltham, MA, USA) according to the manual. Briefly, the cells were crosslinked by 1% formaldehyde and the reaction was quenched by glycine. The cells were then scraped and transferred into a new tube for lysis with lysis buffer. Sonication was employed for DNA fragmentation. Chromatin was collected by centrifugation. KDM4C antibody-, ALDH1A3 antibody-, H3K9me3 antibody-, H3K9me2 antibody-Dynabeads protein A/G were prepared and were used for immunoprecipitation of KDM4C-, ALDH1A3-, H3K9me3-, H3K9me2-linked DNA. Finally, the beads were separated and the DNA linked with beads was purified, followed by qRT-PCR examination.

**Tumourigenicity, in vivo drug sensitivity assay and survival analysis**

For tumourigenesis examination, 5 × 10^6^ cells in 0.2 ml medium-matrigel mixture (3:1) were inoculated into the flank of BALB/c nude mice (n=7). The tumour volume was recorded every 7 days. For drug sensitivity and survival analysis, after inoculation (n=9), the drugs were administrated when the tumour size (length × width^2^/2) reached 140 mm^3^. The tumour size was recorded every 7 days. One representative of each group was sacrificed at the 21^st^ day for electronic record. The sensitization was calculated as 100% × ((Tumour volume_5-FU/cisplatin_ - Tumour volume_(5-FU/cisplatin + knockdown)_) / Tumour volume_5-FU/cisplatin_). The rest mice in each group were used for survival analysis, which were sacrificed when one of following criteria were reached: 1) tumour length ≥ 20 mm; tumour volume ≥ 2,000 mm; 45 days after inoculation. GraphPad PRISM v 6.0 software was used for survival analysis.

**Drug sensitivity assay (in vitro)**

Normal or sphere-derived cells were cultured in 96 well plated (3 × 10^3^ cells per well). After attachment, the culture medium was changed into drug containing medium. After 48 h, the number of cells was examined by CCK-8 assay (Dojindo Molecular Technologies, Tokyo, Japan). The cell viability was calculated as (absorbance_drug_ - absorbance_blank_) / (absorbance_vehicle_ - absorbance_blank_)%. R program was used for calculation of the half-maximal inhibitory concentration (IC50). The sensitization = (viability_drug_ – viability_(drug + knockdown)_) / viability_drug_%.

**Protein degradation test**

The translation of the cells was inhibited by cycloheximide (CHX, Cell Signalling Technology, Inc. Danvers, MA, USA). The total protein of the cells was isolated by RIPA reagent and the protein level of target was analyzed by western blot. The degradation of the target protein = ((blot density_0h_ – blot density_24h_) / (blot density_0h_).

**Cellular fractionation**

Protein fractions were isolated by Subcellular Protein Fractionation kit (Thermo Fisher, Waltham, MA, USA) according to the manual. Briefly, the freshly dissociated cells were lysed by cytoplasmic extraction buffer (CEB) (4 °C, 10 min), followed by centrifugation (500 g, 5 min). The supernatant was collected and transferred into a new tube as cytoplasmic proteins. The pellet was then incubated with membrane extraction buffer (MEB) (4 °C, 10 min), followed by centrifugation (3,000g, 5 min). The pellet was then maintained in IB sample buffer and boiled (5 min) to extract nuclear proteins.

**Statistics**

The data are presented as mean ± SD (n=3 independent experiments with 3 technical repeats). Student’s t‐test (two-tailed) and one-way ANOVA were used for P value calculation. P < 0.05 was considered as statistical significance.

**Reference:**

1. Specht, E, et al. Comparison of immunoreactive score, HER2/neu score and H score for the immunohistochemical evaluation of somatostatin receptors in bronchopulmonary neuroendocrine neoplasms. *Histopathology* **67**, 368 (2015).

**Supplementary Table S1. Primers used in this study.**

| **Reverse transcription PCR** | |
| --- | --- |
| KDM4C | F: 5’- ATGGAGGTGGCCGAGGTGGAAAGTCC-3’ |
|  | R: 5’-CTAGATTCCCAGCCTTCCCAATTTT-3’ |
| ALDH1A3 | F: 5’- ATGGCCGCCGCTAACGGGGCCGTGGAA-3’ |
|  | R: 5’- TCAGGGGTTCTTGTCGCCAAGTTTGA-3’ |
| KDM4C promoter | F: 5’- TTAAAGTGGATTCAAAAATATTT-3’ |
|  | R: 5’- CAAACATCCTTTAGCCTCTTCCAC-3’ |
| ALDH1A3 promoter | F: 5’-CGCCAGTGTTAGCCAGCCGATATCGG-3’ |
|  | R: 5’-GGCTCCTCCGCGCTCCCTGGCCCGAGG-3’ |
| Real-Time Quantitative Reverse Transcription PCR | |
| KDM4C | F: 5’-GATGAATGGAACATAGCTCGCC-3’ |
|  | R: 5’-GGTGTGCCATGCAAACGTG-3’ |
| CD44 | F: 5’-CTGCCGCTTTGCAGGTGTA-3’ |
|  | R: 5’-CATTGTGGGCAAGGTGCTATT-3’ |
| SOX2 | F: 5’- GCCGAGTGGAAACTTTTGTCG |
|  | R: 5’-GGCAGCGTGTACTTATCCTTCT |
| OCT4 | F: 5’-CTTGAATCCCGAATGGAAAGGG |
|  | R: 5’-GTGTATATCCCAGGGTGATCCTC |
| CD133 | F: 5’-AGTCGGAAACTGGCAGATAGC |
|  | R: 5’-GGTAGTGTTGTACTGGGCCAAT |
| ALDH1A3 | F: 5’-5'- ACCTCTCACCGCCCTTTATCT-3' |
|  | R: 5'-GTGAAGGCGATCTTGTTGATCT-3' |
| KDM4C promoter | F: 5’-CATTGTTTCTTGGAACCACAATTG-3’ |
|  | R: 5’-CAAACATCCTTTAGCCTCTTCCAC-3’ |
| ALDH1A3 promoter | F: 5’-GCGTGGGGCGCTGCATAAAGCGG-3’ |
|  | R: 5’-GGCTCCTCCGCGCTCCCTGGCCCGAGG-3’ |
| GAPDH | F: 5’-CTGGGCTACACTGAGCACC-3’ |
|  | R: 5’-AAGTGGTCGTTGAGGGCAATG-3’ |
| PDX | F: 5’-GGAGCAGGATTGTGCCGTAA-3’ |
|  | R: 5’-CTGTGGGGACGCACTAAGG-3’ |
| SLC9A3R1 | F: 5’-GGCTGGCAACGAAAATGAGC-3’ |
|  | R: 5’-TGTCGCTGTGCAGGTTGAAG-3’ |
| HSPA4 | F: 5’-AGTGATGGATGCAACACAGATT-3’ |
|  | R: 5’-CCAATGTCGTGTCAAATGCAG-3’ |
| MYH14 | F: 5’-CGGAACACCGATCAAGCCA-3’ |
|  | R: 5’-CGGGAGAAATCCGTCACCC-3’ |
| PPP2R2A | F: 5’-CATACCAGGTGCATGAATACCTC-3’ |
|  | R: 5’-GGGTTATGTCTCGCTTTGTGTTT-3’ |
| MYH9 | F: 5’-CAGCAAGCTGCCGATAAGTAT-3’ |
|  | R: 5’-CTTGTCGGAAGGCACCCAT-3’ |
| ACTN4 | F: 5’-GCAGCATGGGCGACTACAT-3’ |
|  | R: 5’-TTGAGCCCGTCTCGGAAGT-3’ |
| ACTN1 | F: 5’-TCCATCGGAGCCGAAGAAATC-3’ |
|  | R: 5’-GTGTCGGTGGATCAAAGCACA-3’ |
| ITGB1 | F: 5’-CCTACTTCTGCACGATGTGATG-3’ |
|  | R: 5’-CCTTTGCTACGGTTGGTTACATT-3’ |
| RDX | F: 5’-TATGCTGTCCAAGCCAAGTATG-3’ |
|  | R: 5’-CGCTGGGGTAGGAGTCTATCA-3’ |
| VCL | F: 5’-CTCGTCCGGGTTGGAAAAGAG-3’ |
|  | R: 5’-AGTAAGGGTCTGACTGAAGCAT-3’ |
| SSH3 | F: 5’-CCTGCTGGTAGTTTCTACACG-3’ |
|  | R: 5’-CACCTGGGTGTCACTCCAGA-3’ |
| BRAF | F: 5’-AATACACCAGCAAGCTAGATGC-3’ |
|  | R: 5’-AATCAGTTCCGTTCCCCAGAG-3’ |
| PFN1 | F: 5’-GGGTGGAACGCCTACATCG-3’ |
|  | R: 5’-CCATTCACGTAAAAACTTGACCG-3’ |
| RIPK2 | F: 5’-CGCTGCTCGACAGTGAAAGAA-3’ |
|  | R: 5’-GCAGGATGCGAAATCTCAATGG-3’ |
| OAS2 | F: 5’-CTCAGAAGCTGGGTTGGTTTAT-3’ |
|  | R: 5’-ACCATCTCGTCGATCAGTGTC-3’ |
| STAT1 | F: 5’-CAGCTTGACTCAAAATTCCTGGA-3’ |
|  | R: 5’-TGAAGATTACGCTTGCTTTTCCT-3’ |
| VDAC2 | F: 5’-GGCGTGGAATTTTCAACGTCC-3’ |
|  | R: 5’-AGACCATACTCACACCACTTGTA-3’ |
| OAS3 | F: 5’-GAAGGAGTTCGTAGAGAAGGCG-3’ |
|  | R: 5’-CCCTTGACAGTTTTCAGCACC-3’ |
| HSP90AB1 | F: 5’-AGAAATTGCCCAACTCATGTCC-3’ |
|  | R: 5’-ATCAACTCCCGAAGGAAAATCTC-3’ |
| DNM1L | F: 5’-CTGCCTCAAATCGTCGTAGTG-3’ |
|  | R: 5’-GAGGTCTCCGGGTGACAATTC-3’ |
| FLNA | F: 5’-CTTATCGCGCTGTTGGAGGT-3’ |
|  | R: 5’-GCCACCGACACGTTCTCAA-3’ |
| FLNB | F: 5’-GTGAACAAACGCATCGGCAA-3’ |
|  | R: 5’-ACCAGACCCAAGATGAGCTTC-3’ |
| TPI1 | F: 5’-CTCATCGGCACTCTGAACG-3’ |
|  | R: 5’-GCGAAGTCGATATAGGCAGTAGG-3’ |
| FBP1 | F: 5’-CGCGCACCTCTATGGCATT-3’ |
|  | R: 5’-TTCTTCTGACACGAGAACACAC-3’ |
| PFKP | F: 5’-GCATGGGTATCTACGTGGGG-3’ |
|  | R: 5’-CTCTGCGATGTTTGAGCCTC-3’ |
| PGK1 | F: 5’-TGGACGTTAAAGGGAAGCGG-3’ |
|  | R: 5’-GCTCATAAGGACTACCGACTTGG-3’ |
| EPHX1 | F: 5’-CTTTGCCATCTACTGGTTCATCT-3’ |
|  | R: 5’-TCTCCTCATCTGACGTTTCCA-3’ |
| AKR1C1 | F: 5’-TTCATGCCTGTCCTGGGATTT-3’ |
|  | R: 5’-CTGGCTTTACAGACACTGGAAAA-3’ |
| SCD | F: 5’-TCTAGCTCCTATACCACCACCA-3’ |
|  | R: 5’-TCGTCTCCAACTTATCTCCTCC-3’ |
| HLA-DPB1 | F: 5’-ACGGTCAAGACCAAGGATGG-3’ |
|  | R: 5’-AGCGTGTATTTCCGCGTGA-3’ |

**Supplementary Table S2. Antibody used in this study.**

| **Antigen** | **Antibody** | **Conjugated with** | **Application** | **Manufacture** | **Catalog #** | **Dilution (1:)** |
| --- | --- | --- | --- | --- | --- | --- |
| KDM4C | Rabbit, IgG | Unconjugated | WB/ChIP/IHC | Abcam | Ab226480 | 1000 for WB, 400 for IHC |
| ALDH1A3 | Rabbit, IgG | Unconjugated | WB/ChIP | Abcam | Ab129815 | 1000 for WB, 400 for IHC |
| GAPDH | Rabbit, IgG | Unconjugated | WB | CST | #5174 | 1000 |
| PU.1 | Rabbit, IgG | Unconjugated | WB | CST | #2266 | 1000 |
| CD44 | Rabbit, IgG | Unconjugated | IHC | CST | #37259 | 400 |
| CD133 | Rabbit, IgG | Unconjugated | IHC | CST | #64326 | 400 |
| SOX2 | Rabbit, IgG | Unconjugated | IHC | CST | #3579 | 300 |
| OCT4 | Rabbit, IgG | Unconjugated | IHC | CST | #2890 | 300 |
| Rabbit IgG | Goat, NA | HRP | WB | CST | 7074 | 2000 |

CST: Cell Signaling Technology, WB: Western blot, HRP: horseradish peroxidase, IHC: Immunohistochemical staining.


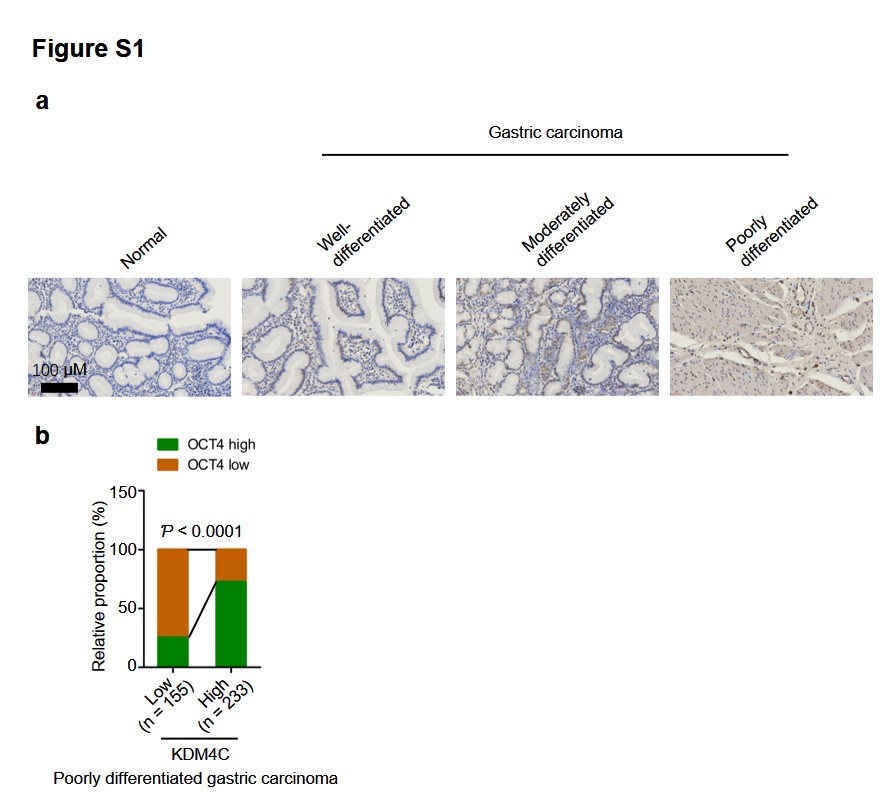


**Figure S1. KDM4C is upregulated in poorly differentiated gastric cancer cells and positively correlated with markers of gastric cancer stem cells.** (**a)** KDM4C immunohistochemical staining in normal, well differentiated, moderately differentiated and poorly differentiated tumor tissues. (**b)** The correlations of KDM4C with OCT4 were determined by Spearman correlation analysis.


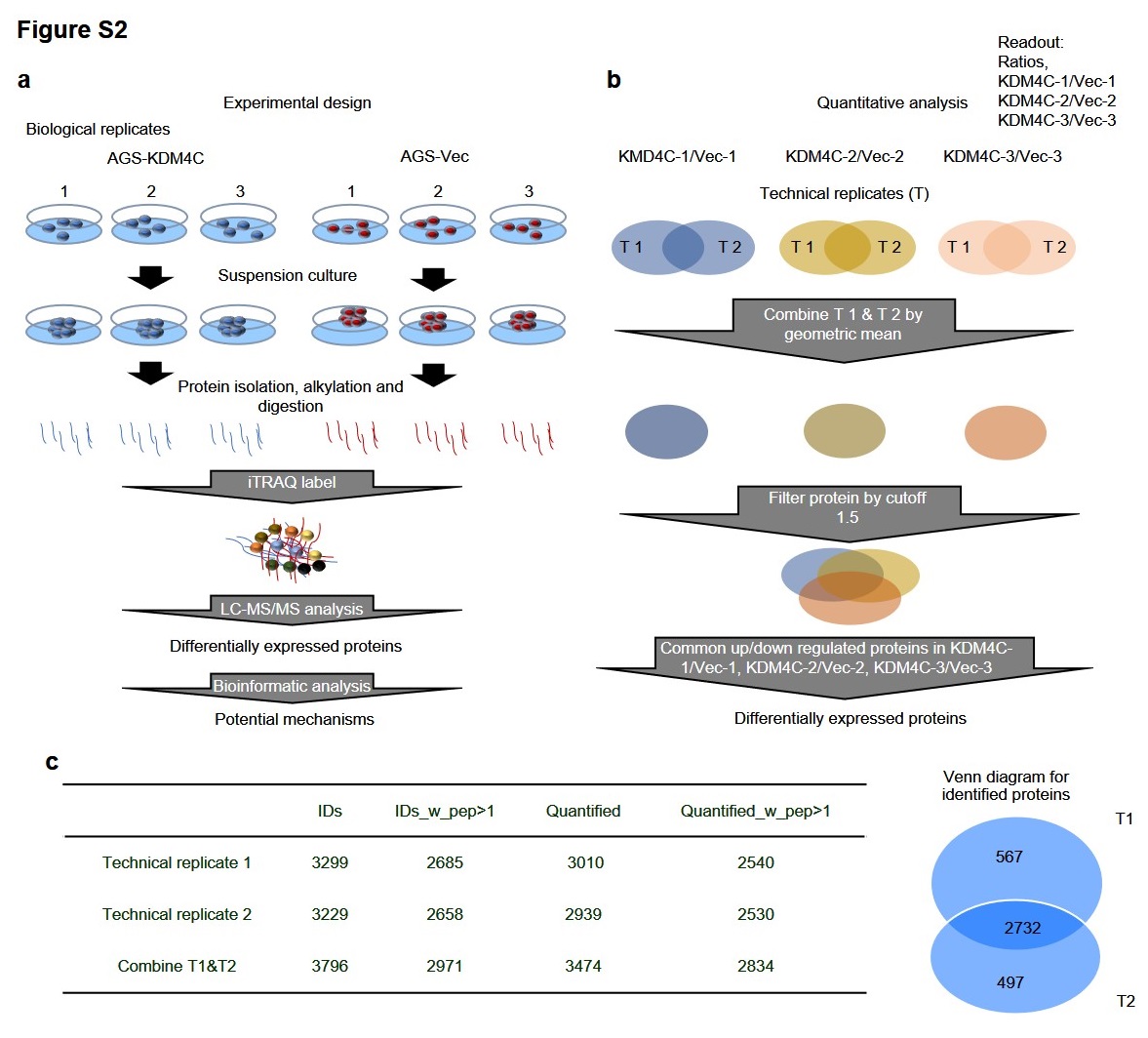


**Figure S2. Proteomic analysis of KDM4C-induced change in proteome of AGS gastric cancer cells. (a,b)** Workflow of biological experiments **(a)** and data analysis **(b)**. The total proteins isolated from KDM4C-overexpressing (AGS-KDM4C) and control (AGS-Vec) cells were subjected to LC-MS/MS analysis, followed by data analysis. In total, 3 biological and 2 technical replicates were performed. **(c)** The results of LC-MS/MS analysis. a total of 3796 proteins were identified (false discovery rate < 1%), 2971 proteins identified with at least two fragments and 3474 proteins were quantifiable.

**
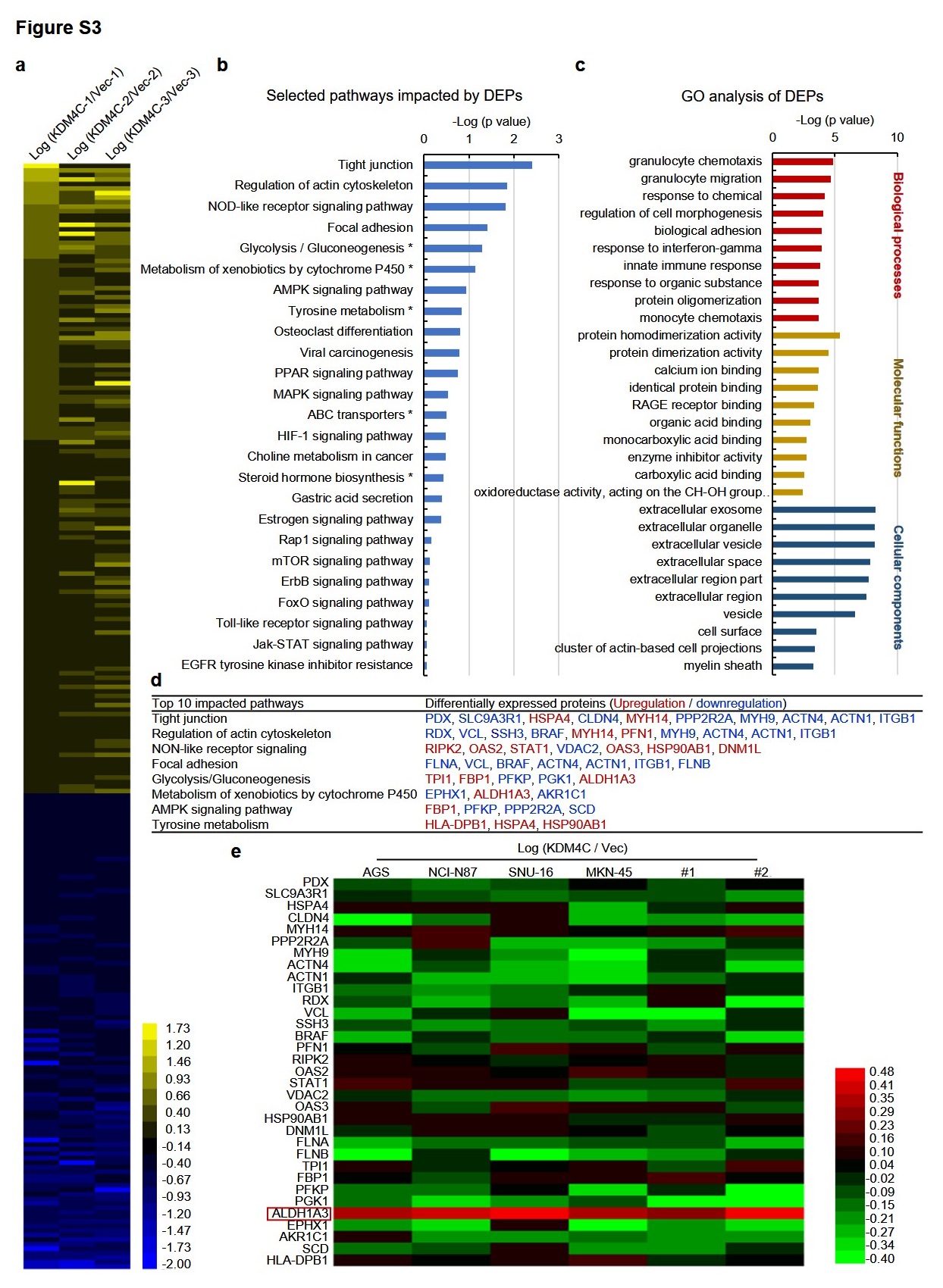
**

**Figure S3. ALDH1A3 is the potential downstream target of KDM4C. (a)** Differentially expressed proteins in KDM4C-overepxressing AGS cells. By comparing the quantified proteins, 244 differentially expressed proteins were identified in KDM4C-overepxressing AGS cells, with 139 proteins upregulated and 105 proteins downregulated. **(b,c)** Pathway **(b)** and GO (Gene Ontology) **(c)** analysis of differentially expressed proteins by iPathwayGuide online software. **(d)** The top 8 impacted pathways and associated differentially expressed genes in KDM4C-overexpressing cells identified by proteomics study. **(e)** Verification of regulation of differentially expressed genes in KDM4C-overexpressing gastric cancer cells. The fold changes of mRNA level (examined by quantitative real-time PCR (Q-PCR)) of indicated genes in KDM4C-overexpressing cell lines (AGS, NCI-N87, SNU-16, MKN-45) and primary (#1, #2) gastric cancer cells were presented as heatmap. Three independent experiments were performed. Only KDM4C-induced upregulation of ALDH1A3 was conserved in all gastric cancer cells.


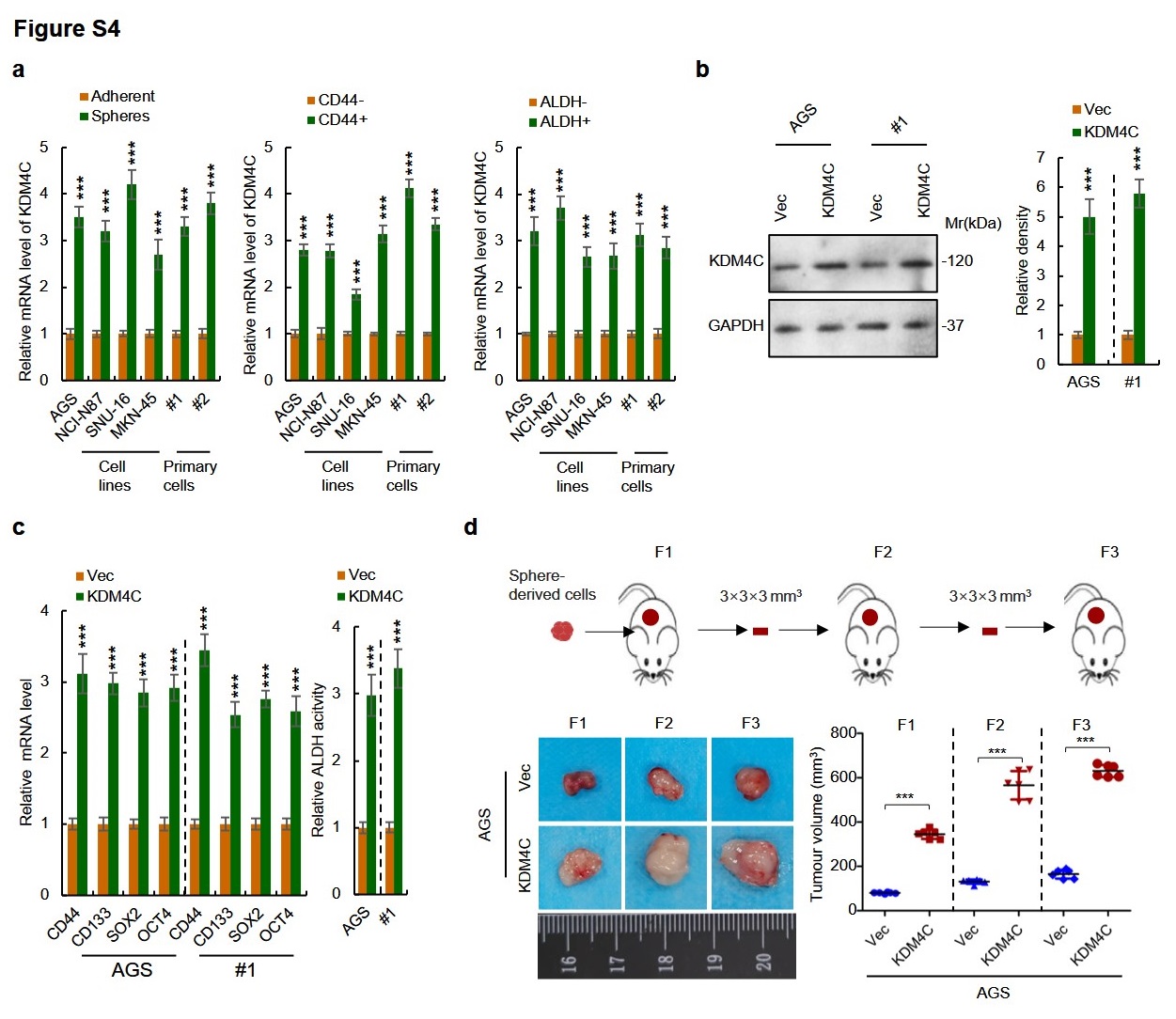


**Figure S4. KDM4C is critical for stemness maintenance of gastric cancer cells.** (**a**) The mRNA levels of KDM4C in indicated gastric cancer cells were analyzed by Q-PCR. Error bars indicate standard deviation (SD) (Student *t* test, n = 3). (**b**) Characterization of KDM4C-overexpressing cells by western blot. Error bars indicate SD (Student *t* test, n = 3). (**c**) The mRNA levels of gastric cancer stem cell markers and ALDH activity in indicated cells were determined Q-PCR and ALDEFLUOR™ Kit, respectively. Error bars indicated SD (Student *t* test, n = 3)**. (d)** The multi-generational tumourigenicity of KDM4C-overexpressing and control AGS cells was examined by xenograft mice model. Error bars indicated SD (n = 6). Student’s *t* test. **Ƥ* < 0.05, ** *Ƥ* < 0.01, *** *Ƥ* < 0.001.

**
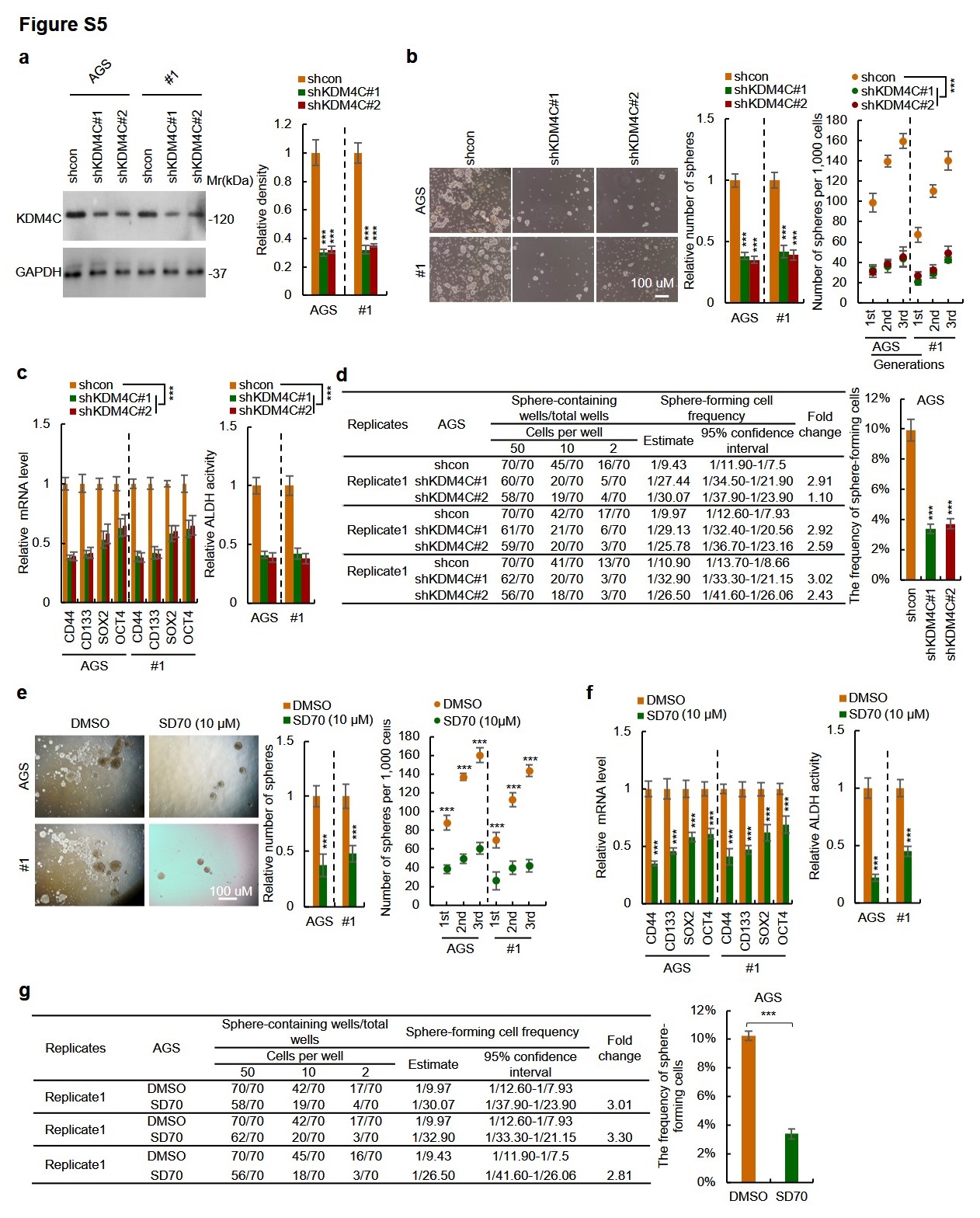
**

**Figure S5. Suppression of KDM4C inhibits the stemness of gastric cancer cells.** (**a**) Characterization of KDM4C-knockdown cells by western blot. Error bars indicate standard deviation (SD). (One-Way ANOVA, n = 3). (**b**) The sphere number of indicated cells at the density of 3,000 cells/well was recorded (left). Error bars indicate SD. The sphere number of primary, secondary, and tertiary passaged KDM4C-overexpressing and control indicated cells was counted (right). The data were analyzed by one-way ANOVA. Error bars indicated SD (n = 3). (**c**) The mRNA levels of gastric cancer stem cell markers and ALDH activity in indicated cells were determined Q-PCR and ALDEFLUOR™ Kit, respectively. The data were analyzed by one-way ANOVA (n = 3). Error bars indicated SD. (**d**) The in vitro self-renewal capacities of indicated cells were determined by limiting dilution assay (one-way ANOVA). Error bars indicated SD (n = 3). (**e-g**) The serial sphere-forming capacity **(e)**, mRNA levels of gastric cancer stem cells **(f)**, ALDH activity **(f)** and the in vitro sphere-forming frequency **(g)** of the cells treated with KDM4C inhibitor were examined. The data were analyzed by Student’s *t* test (n = 3). Error bars indicated SD. **Ƥ* < 0.05, ** *Ƥ* < 0.01, *** *Ƥ* < 0.001.

**
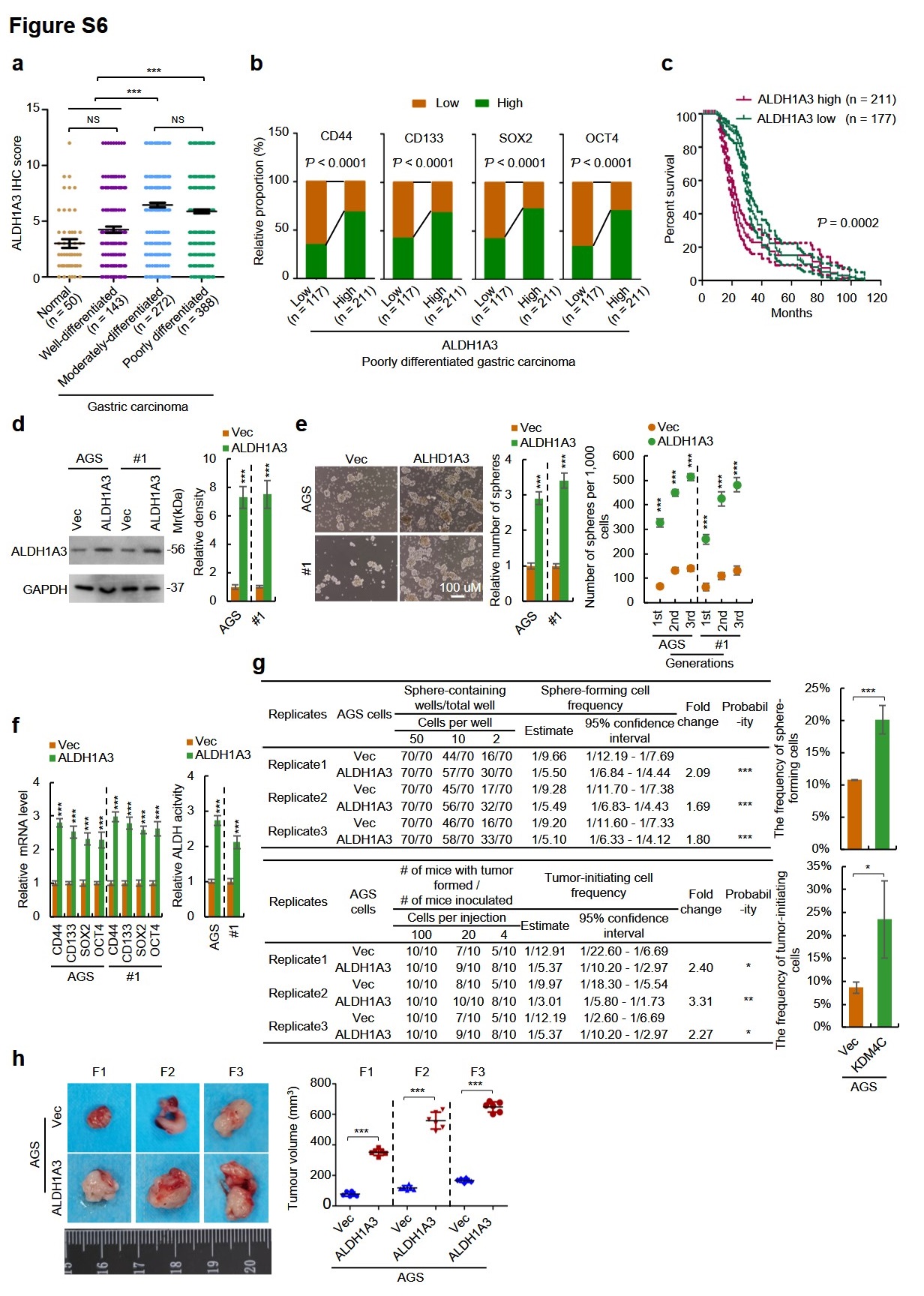
Figure S6. ALDH1A3 promotes gastric cancer stemness.** **(a)** The scores of ALDH1A3 immunohistochemical staining in 50 normal 143 well differentiated, 272 moderately differentiated and 388 poorly differentiated tumor tissues. The data were analyzed by one-way ANOVA. Error bars indicate standard error of the mean. **(b)** The correlations of ALDH1A3 with CD44, CD133, SOX2 and OCT4 were determined by Spearman correlation analysis. **(c)** The association of ALDH1A3 expression with overall survival of poorly differentiated gastric cancer patients was analyzed by the Kaplan-Meier analysis and 95% confidence interval was shown. **(d)** Characterization of ALDH1A3-overexpressing cells by western blot. Error bars indicate standard deviation (SD) (Student *t* test, n = 3). **(e)** ALDH1A3 promotes the sphere-forming capacity of gastric cancer cells on serial passage. The sphere number of indicated cells at the density of 3,000 cells/well was recorded (left). Error bars indicate SD (n = 3). The sphere number of primary, secondary, and tertiary passaged KDM4C-overexpressing and control indicated cells was counted (right). The data were analyzed by Student’s *t* test. Error bars indicate SD (n = 3). **(f)** The mRNA levels of gastric cancer stem cell markers and ALDH activity in indicated cells were determined Q-PCR and ALDEFLUOR™ Kit, respectively. The data were analyzed by Student’s *t* test. Error bars indicate SD (n = 3). **(g)** The in vitro (top) and in vivo (bottom) self-renewal capacities of ALDH1A3-overexpressing and control gastric cancer cells were determined by limiting dilution assay (Student’s *t* test). Error bars indicate SD (n = 3). **(h)** The multi-generational tumourigenicity of ALDH1A3-overexpressing and control AGS cells was examined by xenograft mice model (Student’s *t* test). Error bars indicate SD (n = 6). **Ƥ* < 0.05, ** *Ƥ* < 0.01, *** *Ƥ* < 0.001.


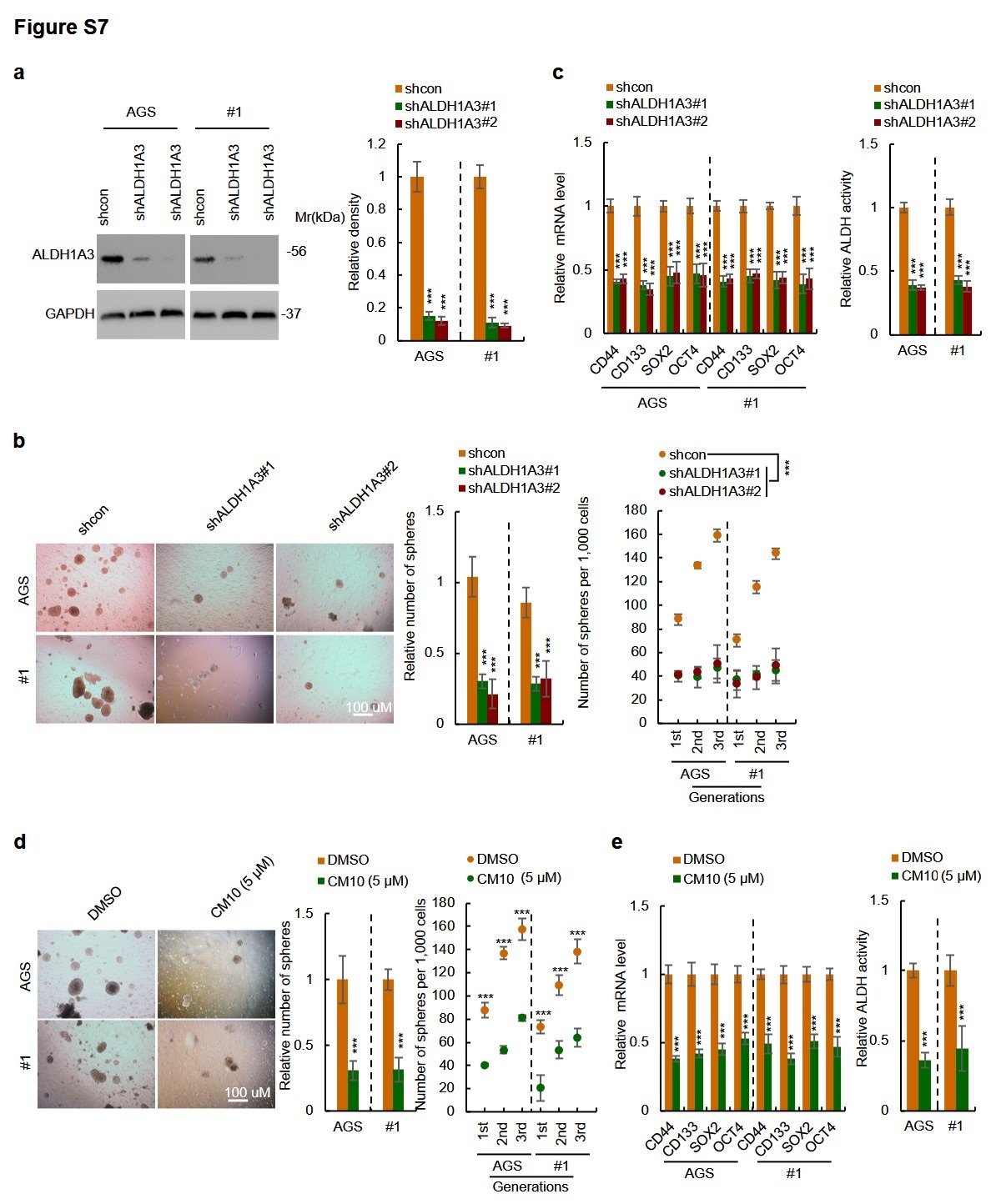


**Figure S7. Suppression of ALDH1A3 inhibits the stemness of gastric cancer cells.** (**a**) Characterization of ALDH1A3-knockdown cells by western blot. Error bars indicate standard deviation (SD) (One-way ANOVA, n = 3). (**b,d**) The sphere number of indicated cells at the density of 3,000 cells/well was recorded (left). The sphere number of primary, secondary, and tertiary passaged KDM4C-overexpressing and control indicated cells was counted (right). The data were analyzed by one-way ANOVA (**b**) and Student’s *t* test (**d**). Error bars indicate SD (n = 3). (**c,e**) The mRNA levels of gastric cancer stem cell markers and ALDH activity in indicated cells were determined Q-PCR and ALDEFLUOR™ Kit, respectively. The data were analyzed by one-way ANOVA (**c**) and Student’s *t* test (**e**). Error bars indicate SD (n = 3). **Ƥ* < 0.05, ** *Ƥ* < 0.01, *** *Ƥ* < 0.001.


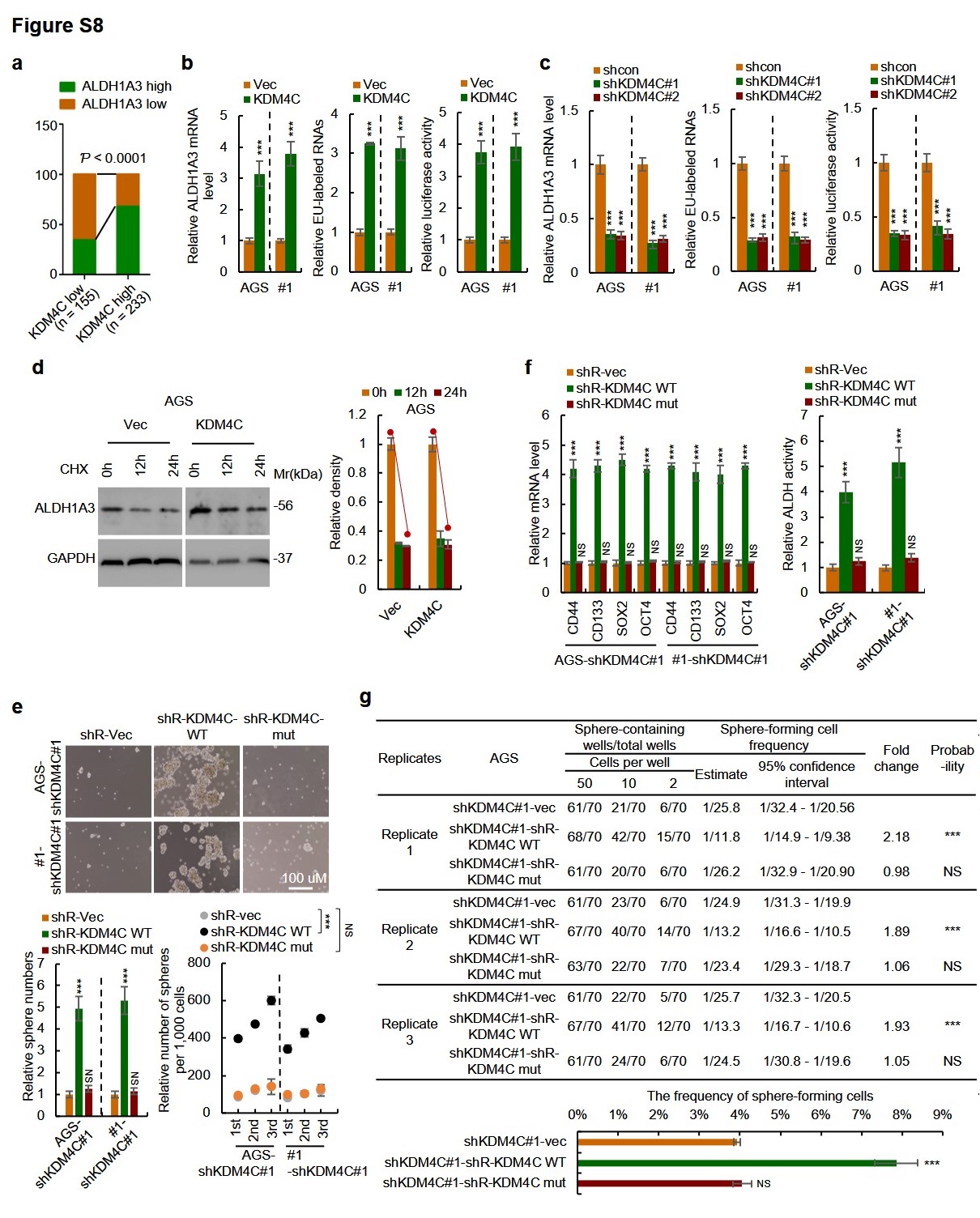


**Figure S8. KDM4C epigenetically activates ALDH1A3 transcription by histone demethylation. (a)** The correlation of KDM4C with ALDH1A3 was determined by Spearman correlation analysis. **(b,c)** The mRNA levels of ALDH1A3 (**left**) in KDM4C-overexpressing (**b**) and KDM4C-knockdown (**c**) cells were analyzed by qRT-PCR. The transcription of ALDH1A3 (**middle**) in KDM4C-overexpressing (**b**) and KDM4C-knockdown (**c**) cells was analyzed by nuclear run-on assay. The transcriptional activities of ALDH1A3 promoter (**right**) in KDM4C-overexpressing (**b**) and KDM4C-knockdown (**c**) cells were analyzed by luciferase reporter assay. The data were analzyed by Student’s *t* test (**b**) and one-way ANOVA **(c).** Error bars indicated standard deviation (SD) (n = 3)**.** (**d**) Western blot analysis of ALDH1A3 protein levels in KDM4C-overexpressing and control AGS cells treated with and without cycloheximide. Error bars indicate SD (n = 3). **(e-g)** Demethylase activity of KDM4C is necessary for KDM4C promoting the stemness of gastric cancer cells. shRNA resistant demethylase dead KDM4C (mutation at H190 and E192) and shRNA resistant wild-type KDM4C were transfected into KDM4C-knockdown AGS and #1 gastric cancer cells. The spherogenicity **(e)**, gastric cancer stem cell markers expression and ALDH activity **(f)** and in vitro sphere-forming capacity **(g)** of the cells were determined. The data were analzyed by one-way ANOVA. Error bars indicated SD (n = 3). **Ƥ* < 0.05, ** *Ƥ* < 0.01, *** *Ƥ* < 0.001.


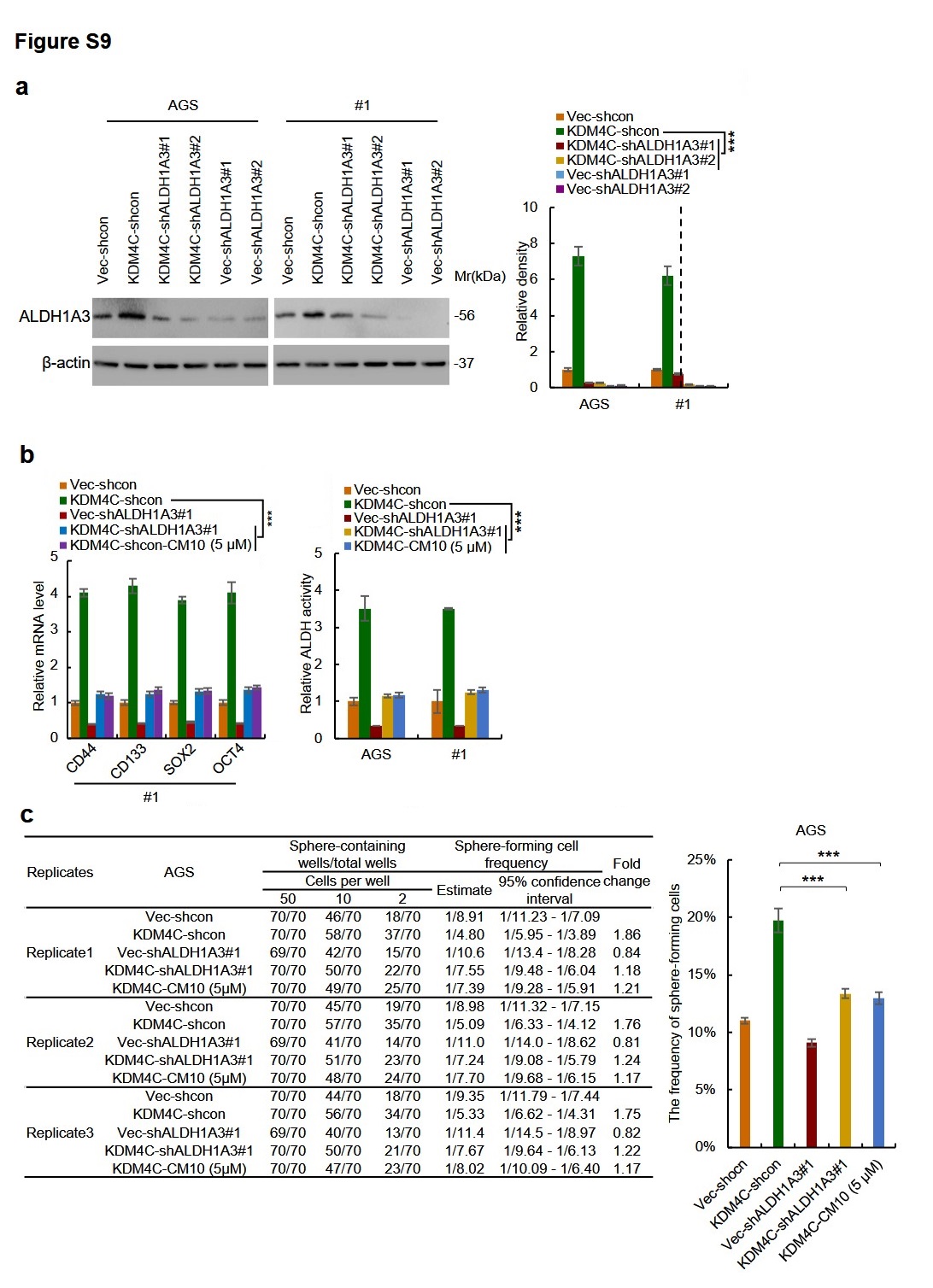


**Figure S9. ALDH1A3 is necessary for KDM4C promoting the stemness of gastric cancer cells. (a)** Characterization of KDM4C-overexpressing ALDH1A3-knockdown cells. Error bars indicate standard deviation (SD) (One-way ANOVA, n = 3). **(b)** The ALDH activity in KDM4C-overexpressing cells with ALDH1A3 depletion or ALDH1A3 inhibitor treatment and control cells were determined**.** Error bars indicate SD (One-way ANOVA, n = 3). **(C)** The frequency of in vitro sphere-forming cells in KDM4C-overexpressing cells with ALDH1A3 depletion or ALDH1A3 inhibitor treatment and control cells were determined**.** The data were analzyed by one-way ANOVA. Error bars indicate SD (n = 3). **Ƥ* < 0.05, ** *Ƥ* < 0.01, *** *Ƥ* < 0.001.


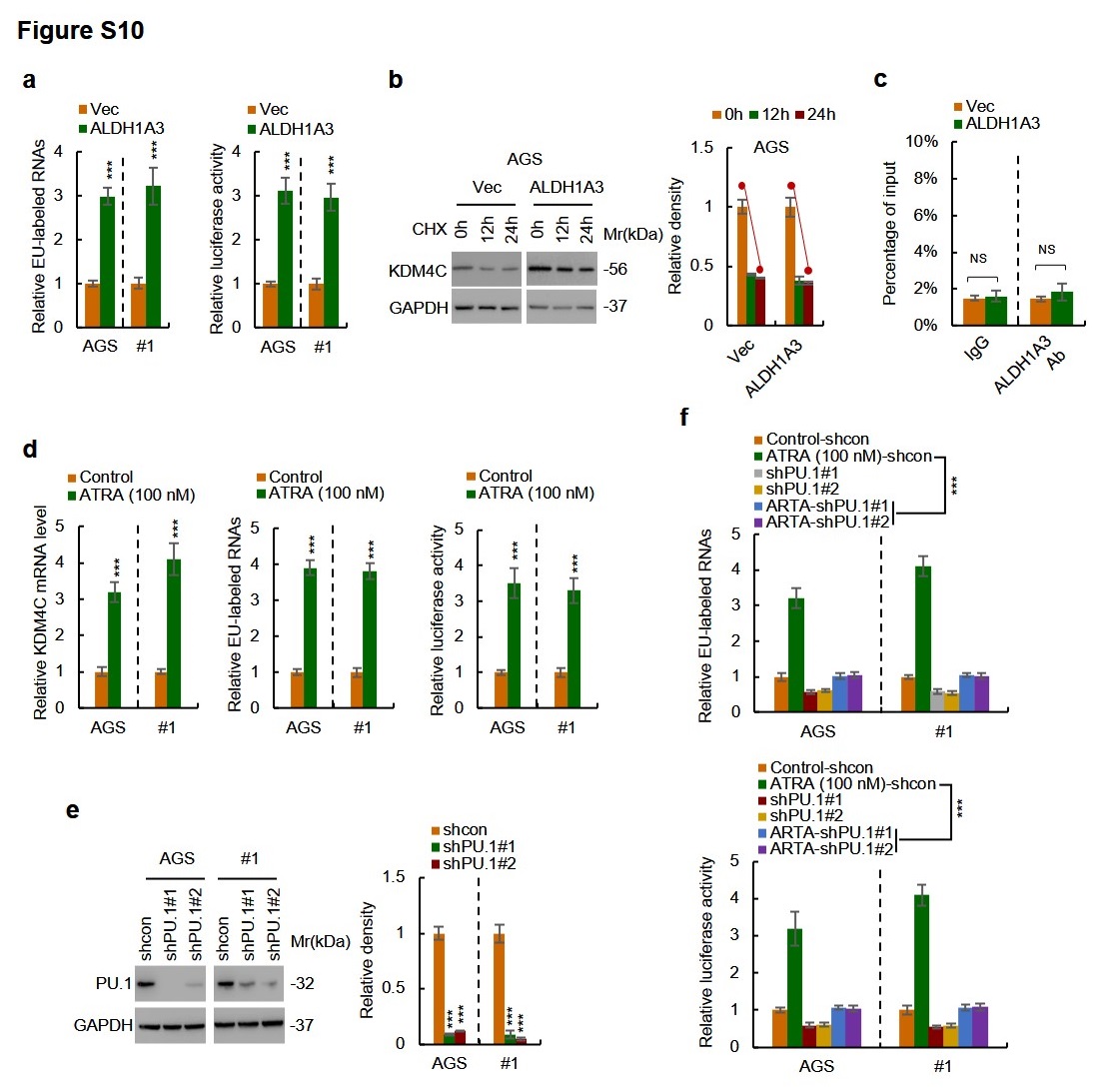


**Figure S10. ALDH1A3 transcriptionally activates of KDM4C by retinoic acid signalling**. **(a)** The transcription of ALDH1A3 (**left**) and the transcriptional activities of ALDH1A3 promoter (**right**) in indicated cells was analyzed by nuclear run-on assay and luciferase reporter assay. The data were analzyed by Student’s *t* test**.** Error bars indicate standard deviation (SD) (n = 3)**. (b)** Western blot analysis of ALDH1A3 protein levels in KDM4C-overexpressing and control AGS cells treated with and without cycloheximide. Error bars indicate standard deviation SD (n = 3) **(c)** The binding between ALDH1A3 and KDM4C promoter was examined by Chromatin immunoprecipitation (ChIP) assay. Error bars indicate SD (Student’s *t* test, n = 3) **(d)** The KDM4C mRNA level (**left**), KDM4C transcription (**middle**) and the transcriptional activity (**right**) of KDM4C promoter in ATRA-treated cells were analyzed by Q-PCR, nuclear run-on assay and luciferase reporter assay (Student’s *t* test). Error bars indicate SD (n = 3)**. (e)** Western blot analysis of the protein level of PU.1 in PU.1-knockdown cells. Error bars indicate SD (One-way ANOVA, n = 3). **(f)** The KDM4C transcription (**left**) and the transcriptional activity (**right**) of KDM4C promoter in indicated cells were analyzed by nuclear run-on assay and luciferase reporter assay. Error bars indicate SD (one-way ANOVA, n = 3). **Ƥ* < 0.05, ** *Ƥ* < 0.01, *** *Ƥ* < 0.001.

**
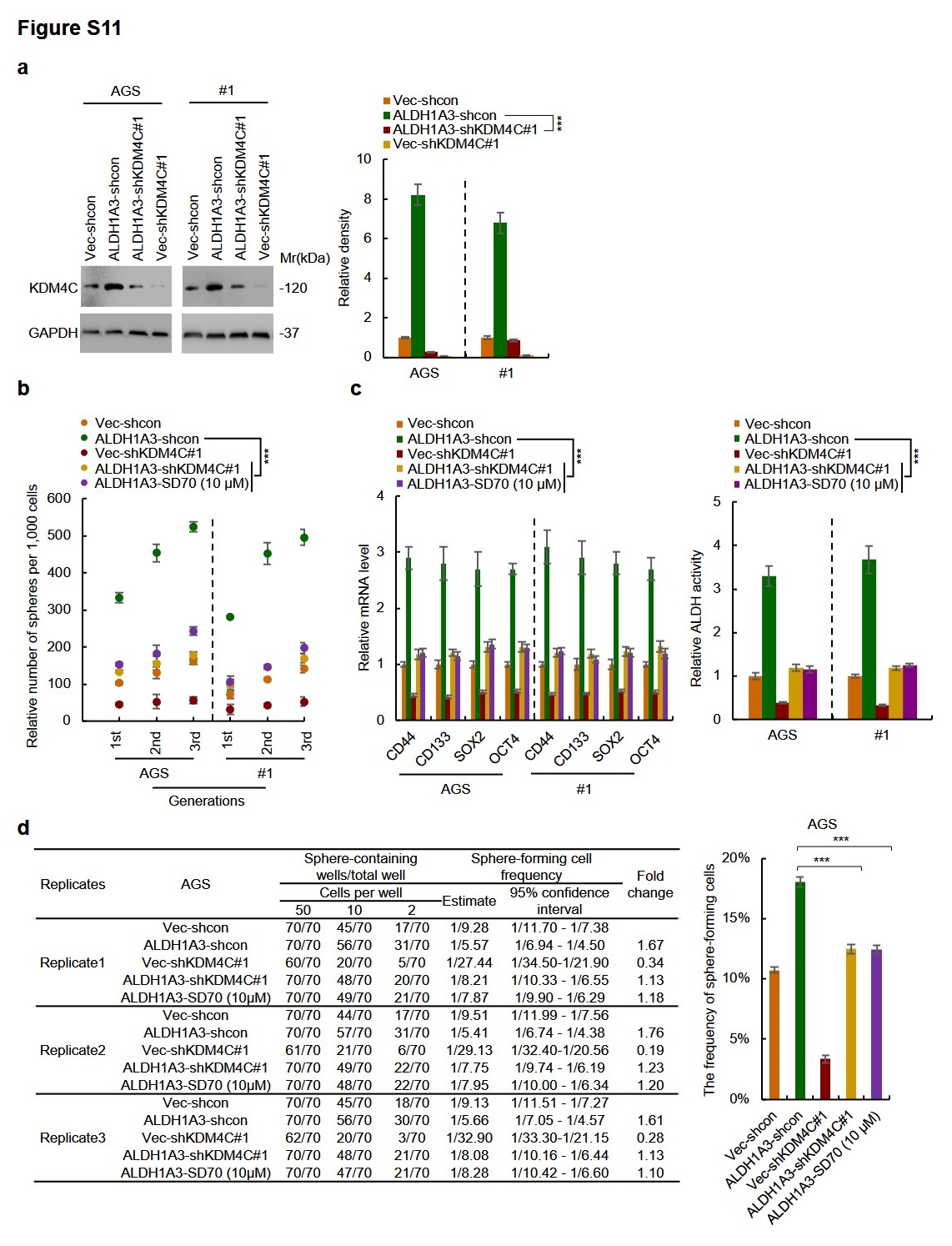
**

**Figure S11. KDM4C is necessary for ALDH1A3 promoting the stemness of gastric cancer cells. (a)** Characterization of ALDH1A3-overexpressing KDM4C-knockdown cells. Error bars indicate standard deviation (SD) (one-way ANOVA, n = 3). **(b-d)** The serial sphere-forming capacity (**b**), the mRNA levels of gastric cancer stem cell markers and the ALDH activity **(c)** and the frequency of in vitro sphere-forming cells **(d)** of the ALDH1A3-overexpressing cells with KDM4C depletion or KDM4C inhibitor treatment and control cells were determined**.** Error bars indicate SD (one-way ANOVA, n = 3). **Ƥ* < 0.05, ** *Ƥ* < 0.01, *** *Ƥ* < 0.001.


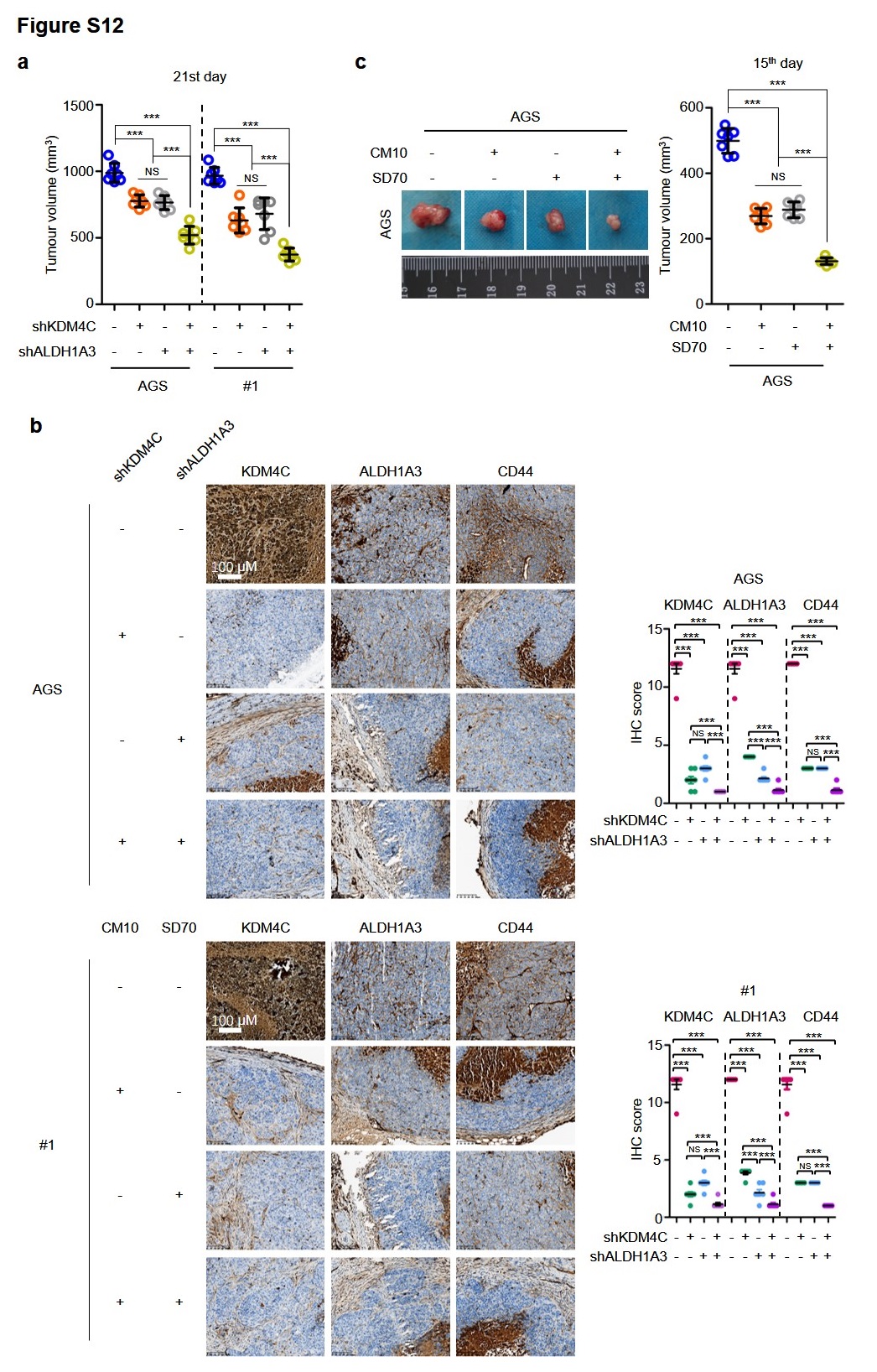


**Figure S12. Inhibition of KDM4C and ALDH1A3 synergistically inhibits tumourignicity of sphere-derived gastric cancer cells. (a)** Inhibition of KDM4C and ALDH1A3 synergistically inhibits tumourigenesis of sphere-derived gastric cancer cells. The tumour volume in mice bearing KDM4C-knockdown, ALDH1A3-knockdown, combination of KDM4C- and ALDH1A3-knockdown and control sphere-derived gastric cancer cells were measured in indicated days (n = 7) (one-way ANOVA). Error bars indicate standard deviation. **(b)** Immunohistochemical staining of KDM4C, ALDH1A3 and CD44 in indicated tumour tissues. Error bars indicate standard deviation. (one-way ANOVA, n = 7) **(c)** The tumour volume in mice bearing KDM4C inhibitor-treated, ALDH1A3 inhibitor-treated, combination of KDM4C inhibitor- and ALDH1A3 inhibitor-treated and control sphere-derived gastric cancer cells were measured in indicated days (n=7) (one-way ANOVA). Error bars indicate standard deviation . **Ƥ* < 0.05, ** *Ƥ* < 0.01, *** *Ƥ* < 0.001.


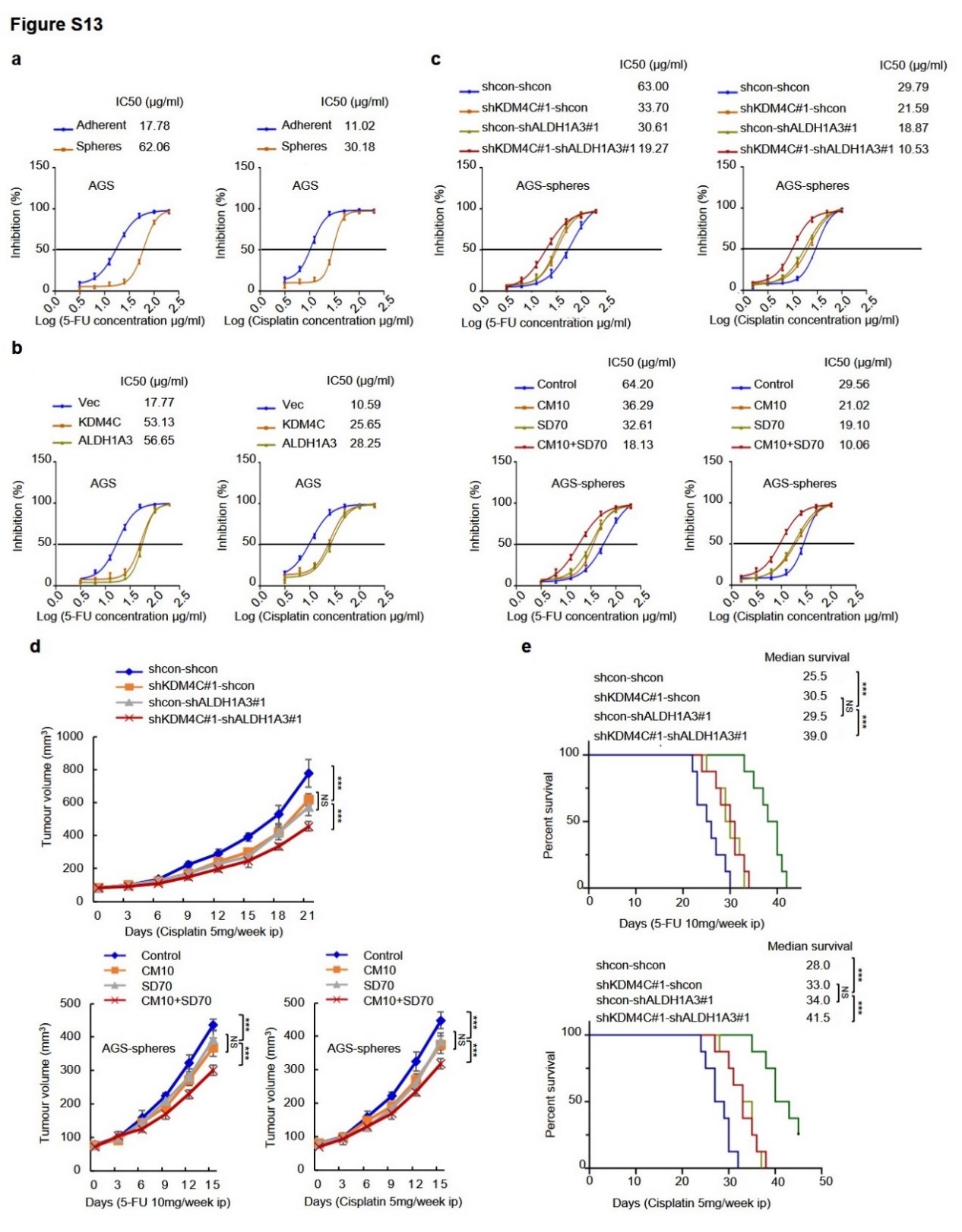


**Figure S13. Inhibition of KDM4C and ALDH1A3 synergistically inhibits chemoresistance of sphere-derived gastric cancer cells. (a)** Sphere-derived gastric cancer cells are more resistant to chemotherapeutic drugs. The half-maximal inhibitory concentration (IC50) of 5-FU and cisplatin in sphere-derived and adherent AGS gastric cancer cells was determined. Error bars indicate standard deviation (SD) (n = 3). **(b)** KDM4C and ALDH1A3 promotes chemoresistance of gastric cancer cells. The IC50 values of 5-FU and cisplatin in KDM4C-overexpressing and ALDH1A3-overexpressing cells and control cells were determined. Error bars indicate SD (n = 3) **(c)** Inhibition of KDM4C and ALDH1A3 synergistically sensitizes gastric cancer cells to chemotherapeutic drugs in vitro. The IC50 values of 5-FU and cisplatin in indicated cells were determined. Error bars indicate SD (n = 3). (**d**) Inhibition of KDM4C and ALDH1A3 synergistically sensitizes gastric cancer cells to chemotherapeutic drugs in vivo. The tumour volume in 5-FU- or cisplatin-treated mice bearing indicated gastric cancer cells was measured in indicated days (n=9). Error bars indicate SD (n = 3) (one-way ANOVA). **(e)** Inhibition of KDM4C and ALDH1A3 synergistically elongated survival of mice bearing sphere-derived gastric cancer cells. The survival of 5-FU- or cisplatin-treated mice bearing indicated sphere-derived gastric cancer cells were recorded (n=8). The data was analyzed by Kaplan-meier analysis. ip: intraperitoneal injection. **Ƥ* < 0.05, ** *Ƥ* < 0.01, *** *Ƥ* < 0.001.
